# Stable inheritance of DNA methylation allows creation of epigenotype maps and the study of epiallele inheritance patterns in the absence of genetic variation

**DOI:** 10.1186/s13059-017-1288-x

**Published:** 2017-08-16

**Authors:** Brigitte T. Hofmeister, Kevin Lee, Nicholas A. Rohr, David W. Hall, Robert J. Schmitz

**Affiliations:** 1 Institute of Bioinformatics, University of Georgia, Athens, GA 30602 USA; 2 Department of Genetics, University of Georgia, Athens, GA 30602 USA

**Keywords:** DNA methylation, Epigenotype, Spontaneous epiallele, Epigenetic map

## Abstract

**Background:**

Differences in DNA methylation can arise as epialleles, which are loci that differ in chromatin state and are inherited over generations. Epialleles offer an additional source of variation that can affect phenotypic diversity beyond changes to nucleotide sequence. Previous research has looked at the rate at which spontaneous epialleles arise but it is currently unknown how they are maintained across generations.

**Results:**

We used two *Arabidopsis thaliana* mutation accumulation (MA) lines and determined that over 99.998% of the methylated regions in the genome are stably inherited across each generation indicating that spontaneous epialleles are rare. We also developed a novel procedure that determines genotypes for offspring of genetically identical parents using only DNA methylation data. The resulting epigenotype maps are highly accurate and strongly agree with expected allele frequency and crossover number. Using epigenotype maps, we explore the inheritance of methylation states in regions of differential methylation between the parents of genetic crosses. Over half of the regions show methylation levels consistent with *cis* inheritance, whereas the other half show evidence of trans-chromosomal methylation and demethylation as well as other possibilities.

**Conclusions:**

DNA methylation is stably inherited by offspring and spontaneous epialleles are rare. The epigenotyping procedure that we describe provides an important first step to epigenetic quantitative trait loci mapping in genetically identical individuals.

**Electronic supplementary material:**

The online version of this article (doi:10.1186/s13059-017-1288-x) contains supplementary material, which is available to authorized users.

## Background

Epigenetic alleles (epialleles) are alleles with differential chromatin states that are mitotically and/or meiotically inherited and are a source of variation that can result in phenotypic diversity. Epialleles have been implicated in numerous phenomena such as hybrid vigor [1–3], genetic incompatibility [4, 5], and stress response (reviewed in [6–8]). Understanding how epialleles are formed and maintained is important for crop improvement, as they represent an untapped source of allelic variation [9, 10].

Epialleles most often arise from changes in cytosine methylation, which is traditionally associated with transcriptional silencing and transposable element (TE) repression. In plants, DNA methylation occurs in three unique sequence contexts: CG, CHG, and CHH where H represents A, C, or T. Each context is maintained by unique pathways and has distinct roles in gene regulation (reviewed in [11]). Genes heavily methylated in all sequence contexts are often transcriptionally silent, whereas only CG methylation in gene bodies (gene body methylation (gbM)) is commonly associated with actively transcribed genes [12, 13].

Spontaneous epialleles occur independent of changes in DNA sequence. Notable examples include the peloric epiallele in toadflax caused by hypermethylation of the *Lcyc* allele [14] and the *COLORLESS NON-RIPENING* (*CNR*) epiallele in tomato caused by hypermethylation of the *cnr* promoter [15]. The mantled phenotype of oil palm is additionally caused by a spontaneous epiallele, resulting in the hypomethylation of a retrotransposon within the *DEFICIENS* gene. This epiallele is of significant agricultural importance, as it results in a substantial loss of yield in affected individuals [16]. Beyond these drastic phenotypes, epialleles also increase phenotypic variation of additional agriculturally important traits such as floral transition [17–20], plant height [18, 19], root length [20], overall crop yield [16, 21], and disease resistance [19, 22]. Collectively, epialleles caused by altered DNA methylation are suggested to be a source of observed missing heritability [23].

Understanding the rate at which epialleles naturally arise continues to be an active area of research. The rate of spontaneous epiallele formation is often confounded by genetic variation, as an epiallele caused by a change in DNA sequence is not truly epigenetic [24, 25]. Previous research involving the use of epigenetic recombinant inbred lines (epiRILs) has greatly increased the understanding of how epialleles are inherited. epiRILs are created using parent plants with the same genetic background, except one parent has a mutation resulting in vastly lowered levels of DNA methylation. Previous studies with epiRILs have shown stable inheritance of DNA methylation over multiple generations and that DNA methylation is mainly additive, although selection against demethylated alleles has been noted [18, 19]. Additionally, epiRILs show extensive phenotypic variation with differentially methylated regions (DMRs) highly associated with altered phenotypes [20, 26–28].

Additional work has taken advantage of different *Arabidopsis thaliana* accessions to explore the inheritance of DNA methylation over generations. After crossing different accessions, known single nucleotide polymorphisms (SNPs) between parents were used to determine chromosomal parent-of-origin and to look for regions of non-additive DNA methylation [29–33]. This strategy has also been applied in crop species such as soybean and corn using recombinant inbred lines [25, 34–36]. These studies showed inherited DNA methylation is mainly additive with rare exceptions, possibly caused by the formation of spontaneous epialleles.

Although the use of epiRILs and differing accessions has greatly expanded the knowledge of how epialleles arise, genetic variation has the capacity to confound results using these two methods. Transposable element reactivation in epiRIL lines as well as genetic variation found in different accessions or inbred lines have the potential to create epialleles which are the result of genetic variation, and thus not truly spontaneous. Previous studies noted that no identified epialleles were near areas of genetic variation; however, this does not eliminate the possibility of *trans*-acting effects or paramutations [25, 36, 37].

An additional approach used to minimize genetic variation is the use of mutation accumulation (MA) lines. Previously, Shaw et al. [38] created a set of *A. thaliana* MA lines from a single Col-0 progenitor. Each line was maintained by single-seed descent for 30 generations [38]. Using whole-genome bisulfite sequencing (WGBS) of early and late generation individuals from MA lines, Schmitz et al. [39] found DNA methylation was consistent for 91% of methylated cytosines. Further analysis has estimated the rate of spontaneous change in DNA methylation at a single cytosine, termed epimutation, to be four to five orders of magnitude greater than nucleotide mutation rate (10^−4^ compared to 10^−9^ per generation per haploid genome, respectively), with losses of DNA methylation more likely than gains [40, 41]. Additionally, epimutations did not accumulate linearly; the number of differentially methylated positions did not increase at a constant rate per generation [41, 42]. However, the number of differentially methylated regions per generation was comparable to the number of nucleotide mutations per generation [39, 42].

Differential methylation of a single cytosine has not been sufficiently linked to altered phenotypes in plants. In contrast, epialleles are known to associate with changes of gene expression and phenotype, but the stability of epialleles through either self-fertilization or outcrossing remains unclear. Furthermore, it is unclear how frequently epialleles arise over sequential generations. In this study, we discover that 99.998% of the methylated regions in the genome were faithfully inherited over generations in two independent MA lines. However, rare spontaneous epialleles were identified and were used to assess the stability of newly formed methylation states using an outcrossing population. As there are not a significant number of nucleotide mutations to distinguish the parental genotypes, a novel epigenotyping method was implemented to determine the parent-of-origin for each of the F2 progeny. Implementing this method revealed that over half of the newly formed parental epialleles segregated in a Mendelian manner. This novel epigenotyping procedure and the resulting data suggest that spontaneous epialleles are sources of allelic variation in crop genomes that are likely stable enough to be used in breeding programs.

## Results

### Transgenerational stability of DNA methylation states

**Fig. 1:**
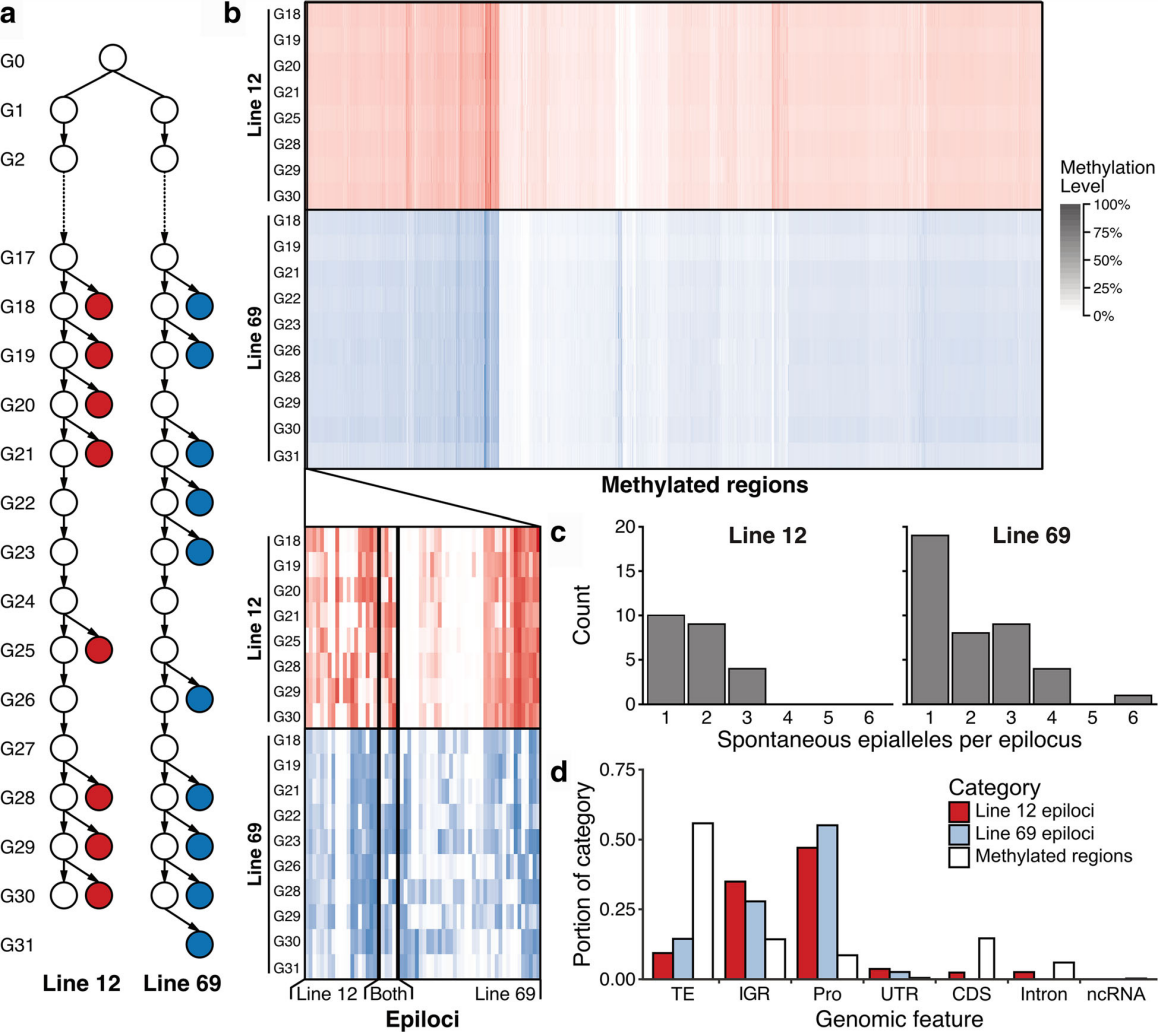
Transgenerational stability of DNA methylation. **a** Experimental set up of MA lines used. *Filled circles* indicate WGBS available. **b** Heatmap showing methylation level of methylated regions in the genome and identified epiallele loci (*inset*) identified in line 12 (n = 19), line 69 (n = 37), or both lines (n = 4). Each column is a region and each row is a sample. Darkness of color indicates methylation level. **c** Distribution of number of novel spontaneous epialleles per epilocus. **d** Distribution of underlying genomic characteristics for all methylated regions (*white*), epialleles in line 12 (*red*), and epialleles in line 69 (*blue*). *Abbreviations*: *TE* transposable element, *IGR* intergenic region, *Pro* promoter, *UTR* untranslated region, *CDS* coding sequence, *ncRNA* non-coding RNA

In previous studies, WGBS was used to assess the inheritance of DNA methylation at individual cytosines over multiple generations [39, 41, 42]. Additionally, previous studies have also explored the spontaneous formation of DMRs between distant generations [39, 42], but it is unknown how stable the newly formed methylation status of these regions is between consecutive generations. To investigate this further, we obtained WGBS data from consecutive generations in two *A. thaliana* Col-0 MA lines [38], line 12 and line 69 (Fig. 1a; Additional file 1: Table S1). Because the majority of the *A. thaliana* genome is unmethylated, we first identified all methylated regions in the genome (methylome). This revealed 23,761 regions (27 Mb) and 21,545 regions (28 Mb) for lines 12 and 69, respectively. We then identified DMRs within each line independently for all generations using differential DNA methylation analysis in all sequence contexts, which revealed 23 DMRs (2157 bp) and 41 DMRs (3981 bp) in lines 12 and 69, respectively (Additional file 1: Table S2). Of the DMRs identified in each line, only four were the same. In these two lines, DMRs constituted less than 0.003% (Fig. 1b) of the methylome and most were specific to each line (Fig. 1b inset).

Next, the stability of the newly formed methylation states at these DMRs was examined. Ten of the 23 DMRs in line 12 and 19 of 41 DMRs in line 69 had a change in DNA methylation states between a single generation (Fig. 1c), indicating a spontaneous epiallele formed that was subsequently faithfully inherited (Additional file 2: Figure S1a). However, 13 regions with multiple changes in DNA methylation states between generations in line 12 and 22 in line 69 were also identified, indicating regions of instability or “hotspots” (Additional file 2: Figure S1b). Overall, DNA methylation states were stably inherited over generations as 99.998 and 99.997% of the methylome identified in lines 12 and 69, respectively, did not contain an epiallele.

Finally, we were interested to know where the epialleles are located in the genome in regards to genomic features (Fig. 1d; Additional file 1: Table S2). Most methylated regions occurred in transposable elements (TEs). However, the majority of the epialleles identified occurred within gene promoters (Pro), defined as 1 kb upstream of the transcription start site. A large portion of epialleles also occurred in intergenic regions (IGRs). Relatively few occurred in TEs and rarely occurred in untranslated regions (UTRs), coding sequences (CDSs), introns, and non-coding RNA (ncRNA) regions (Fig. 1d; Additional file 1: Table S2).

### Inheritance of epialleles upon outcrossing

**Fig. 2:**
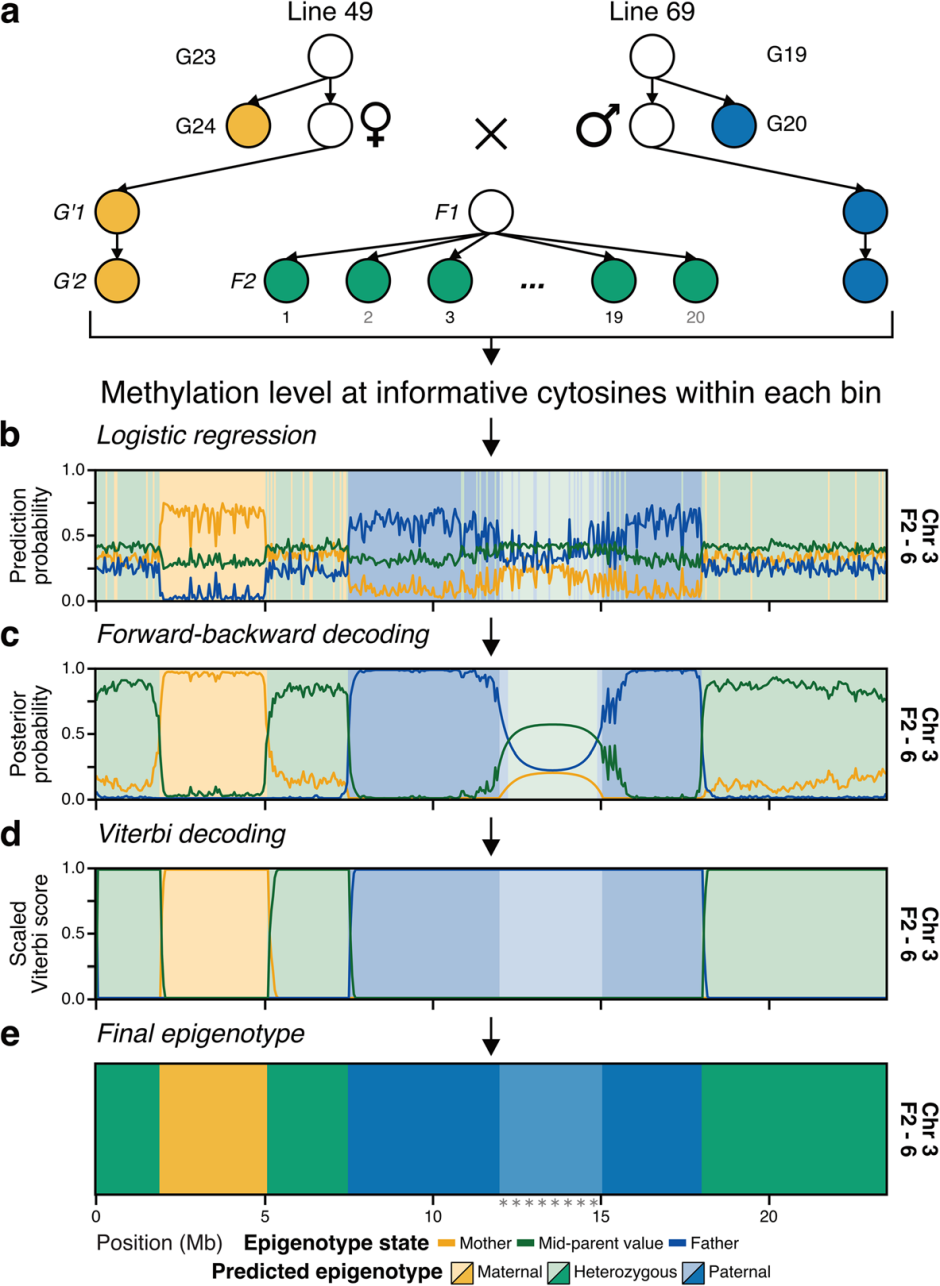
Set-up of MA line 49 × line 69 and overview of epigenotyping procedure. **a** Experimental set up for genetic cross of *A. thaliana* Col-0 MA line 49 × line 69. *Filled circles* indicate WGBS data are available. After obtaining methylation level of all informative cytosines for a bin for all samples, **b** use logistic regression classifier to get initial epigenotype predictions and probabilities for classifiers mother, mid-parent, and father for each bin. **c** Then, apply forward-backward algorithm to find the most likely epigenotype at each bin. **d** Next, apply the Viterbi algorithm to find the most likely sequence of epigenotypes across the chromosome. **d** The resulting map of the Viterbi algorithm is used as the final predicted epigenotype. For **b**–**e**, *background color* indicates epigenotype prediction at each step. *Line* indicates prediction probability, posterior probability from forward-backward algorithm, and scaled score from the Viterbi algorithm. Maternal epigenotype is shown in *yellow*, mid-parent/heterozygous *green*, and paternal *blue*. Centromere is denoted by *gray stars* and *lighter background color*

To help separate genetic effects on the inheritance of spontaneous epialleles during outcrossing, we crossed an individual from line 49 and line 69 of the Shaw et al. MA lines [38] (Fig. 2a). Line 49 generation 24 (G24) was the maternal parent, and line 69 generation 20 (G20) was the paternal parent. From the resulting offspring, the F1 generation, a single random individual was chosen and self-crossed to produce the F2 generation. Twenty F2 individuals were randomly selected for further analysis by WGBS to assess the inheritance of DNA methylation of differentially methylated regions between the two parents. Additionally, both parents were self-crossed to produce the G′1 generation. A random individual from each G′1 population was subsequently self-crossed to produce the G′2 generation. The individuals used in the G′1 crosses and a randomly selected G′2 individual for each line were subjected to WGBS (Fig. 2a; Additional file 1: Table S1).

In previous studies, the ability to study the inheritance of DNA methylation states relied on utilizing genetic variation, often SNPs, in sequenced data to assign regions to each parent, making it possible to compare DNA methylation between parents and progeny. As the MA lines used in this experiment have nearly identical genomes, this approach could not be used, and instead required the development of a method that is not dependent on genetic variation to assign regions to the correct parent in each of the progeny. We hypothesized that if DNA methylation is stably inherited for the vast majority of methylated regions in the genome, DNA methylation data could be used to create a genetic map—termed an epigenotype map [24]. To test this, cytosines in all sequence contexts were identified that were differentially methylated in the parents. Using these informative positions, we then obtained the methylation level [43] of positions sufficiently covered in all samples to be analyzed. Next, each chromosome was split into non-overlapping bins. Bins with less than three informative positions were combined with adjacent bins to minimize the predictive bias of bins with little discriminatory power. The “mid-parent” methylome was computed for all positions in the bin by averaging the methylation level of the parents at each position. When available, additional mid-parent methylomes were created from parental replicates. With the methylation values, a logistic regression classifier was trained for the mother, mid-parent, and father samples. Classification states were weighted by the expected heterozygosity of F2 individuals, 0.25, 0.5, and 0.25 (1:2:1) for mother, mid-parent, and father, respectively. The trained classifier was applied to all samples and the state (classification) with the highest probability at each bin was used as the putative epigenotype (Fig. 2b; Additional file 2: Figure S2).

Crossovers are not expected to occur at high frequencies over small regions of chromosomes, which required the addition of a smoothing method to minimize these likely spurious events. This was accomplished by a forward-backward algorithm, which is commonly used in signal decoding to correct for noise [44]. The algorithm generated posterior probabilities to determine the most likely state at each bin (Fig. 2c). The state with the highest probability was used as the next putative epigenotype (Additional file 2: Figure S3). The centromere and pericentromeric heterochromatin caused difficulties for the logistic regression classifier due to their repetitive nature. The logistic regression classifier predicted the centromere of all samples to be the mid-parent state even when regions just outside of the centromere were classified as mother or father (Additional file 2: Figure S3). To overcome this, the centromere was masked when calculating posterior probability (see “Methods”).

After applying the forward-backward algorithm, the Viterbi algorithm was applied to find the most likely sequence of states across the chromosome (Fig. 2d; Additional file 2: Figure S4) [44]. The centromere region was again masked when applying the Viterbi algorithm. The predicted states from the Viterbi algorithm were used as the final predicted epigenotype (Fig. 2e). Bins predicted as the mother state and the father state represented homozygous maternal and paternal epigenotype, respectively. Bins predicted as the mid-parent state represented the heterozygous epigenotype. Adjacent bins with the same epigenotype represented inherited haplotype blocks and changes in epigenotype between adjacent bins represented crossovers.

### Simulation testing for prediction of epigenotypes

To test the correctness of the proposed procedure, we generated a series of simulated datasets using the parental methylation values of chromosome 3. *A. thaliana* has an average of one to two crossovers per chromosome [45, 46]. It was important to test for increasing number of crossovers to determine potential limits of the procedure. For each simulation iteration, 20 samples were created with zero to 19 potential points of crossover on the chromosome (see “Methods”). Each region of the samples was randomly assigned an epigenotype as maternal homozygous, heterozygous, or paternal homozygous with probabilities 0.25, 0.5, and 0.25 (1:2:1), respectively, as expected by Mendelian inheritance. For each sample, simulated methylomes were created using the parental methylation level of each position within the region. To test the ability of the procedure to handle error around expected methylation levels, additional methylomes were created using randomly generated values within ± 10 to 100% of the parental methylation value. Each set of samples for a given error level was tested with the procedure using six bin sizes of 10 to 500 kb. The final epigenotype prediction was then compared to the assigned epigenotype and accuracy was computed. This process was applied for 25 total iterations (Additional file 2: Figure S5).

**Fig. 3:**
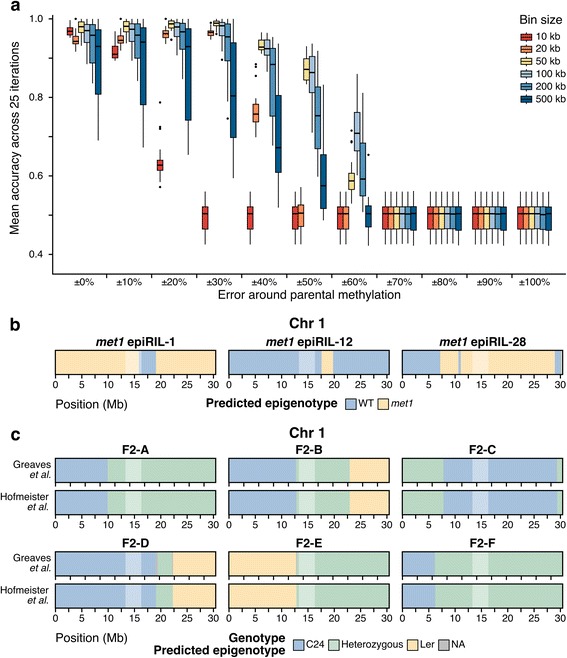
Validation of epigenotyping procedure. **a** Distribution of accuracy for samples tested using different combinations of bin size and error around the parental methylation value. Each data point is the mean accuracy of 25 simulations for a given sample, bin size, and error value. *Dark bar* indicates median, *box* denotes first and third quartile, and *whiskers* are 1.5 times the interquartile range. **b** Epigenotype maps for chromosome 1 of three *met1* epiRIL samples. *Blue* denotes wild type (*WT*)-derived region of chromosome and *yellow* denotes *met1*-derived region. Maps for chromosomes 2-5 are in Additional file 2: Figure S7 **c** Comparison of genetic maps using SNPs created by Greaves et al. [33] to epigenotype maps for chromosome 1. *Blue* denotes C24 homozygous region, *yellow* denotes Ler homozygous region, and *green* denotes heterozygous region as predicted by each method. *Gray* indicates regions where genotype could not be determined. Maps for chromosomes 2–5 are in Additional file 2: Figure S8

The epigenotyping procedure worked well when methylation values were within ±50% of the expected parental methylation values and when using bin sizes greater than 10 kb (Fig. 3a). Larger bin sizes were less accurate for samples with increasing number of crossovers because a bin could potentially span multiple crossovers (Additional file 2: Figure S6). Additionally, larger bin sizes were better able to handle increasing error values than smaller bin sizes (Fig. 3a). Overall, 50-kb bins and ±30% variability had the highest accuracy at 98.69%, validating the utility of the epigenotyping method (Additional file 1: Table S3). Using 50-kb bins was robust to increasing numbers of breakpoints and increasing error around the expected parental methylation (Additional file 2: Figure S6). For these reasons, 50-kb bins were used for further analysis of our genetic cross.

### Epigenotyping of *met1* epiRILs

To test the procedure on existing datasets, epiRILs were subsequently analyzed. In an epiRIL population, each line had a mosaic methylome of normal DNA methylation inherited from the wild type (WT) parent and hypomethylation inherited from the mutant parent, with minimal genetic differences [18, 19]. We used previously published WBGS data for Col-0 WT, Col-0 *met1*-*3*, and F8 generation of three *met1* epiRILs [39, 47, 48] (Additional file 1: Table S1). The class weights were adjusted to account for the expected low residual heterozygosity of F8 individuals. Additionally, the logistic regression classifier was biased towards the WT sample. To overcome this, it was necessary to adjust the input probabilities when applying the forward-backward algorithm (see “Methods”). Due to remethylation events which have been previously documented in the epiRILs [19, 48], using cytosines in all contexts produced ambiguous maps. By using only CG cytosines within gene body methylated genes, which are unlikely to be remethylated [48], we were able to successfully create epigenotype maps for three independent *met1* epiRILs (Fig. 3b; Additional file 2: Figure S7).

### Epigenotyping for C24-Ler crosses

For further validation, we sought to test the procedure on a cross with additional genetic diversity. This would allow us to compare the genetic map created using SNPs to the epigenotype map. We used previously published methylation for a cross between *A. thaliana* accessions C24 and Ler [31] and six F2 samples [33] (Additional file 1: Table S1). The epigenotype map was able to recapitulate the SNP-based genetic map at 99.8% of bins (Fig. 3c; Additional file 2: Figure S8) and was able to predict all recombination events except for one event on chromosome 4 of F2-E that occurred at the heterochromatic knob (Additional file 2: Figure S8). The average difference in breakpoints between the two methods was 18 kb (Additional file 1: Table S4).

### Epigenotype maps

**Fig. 4:**
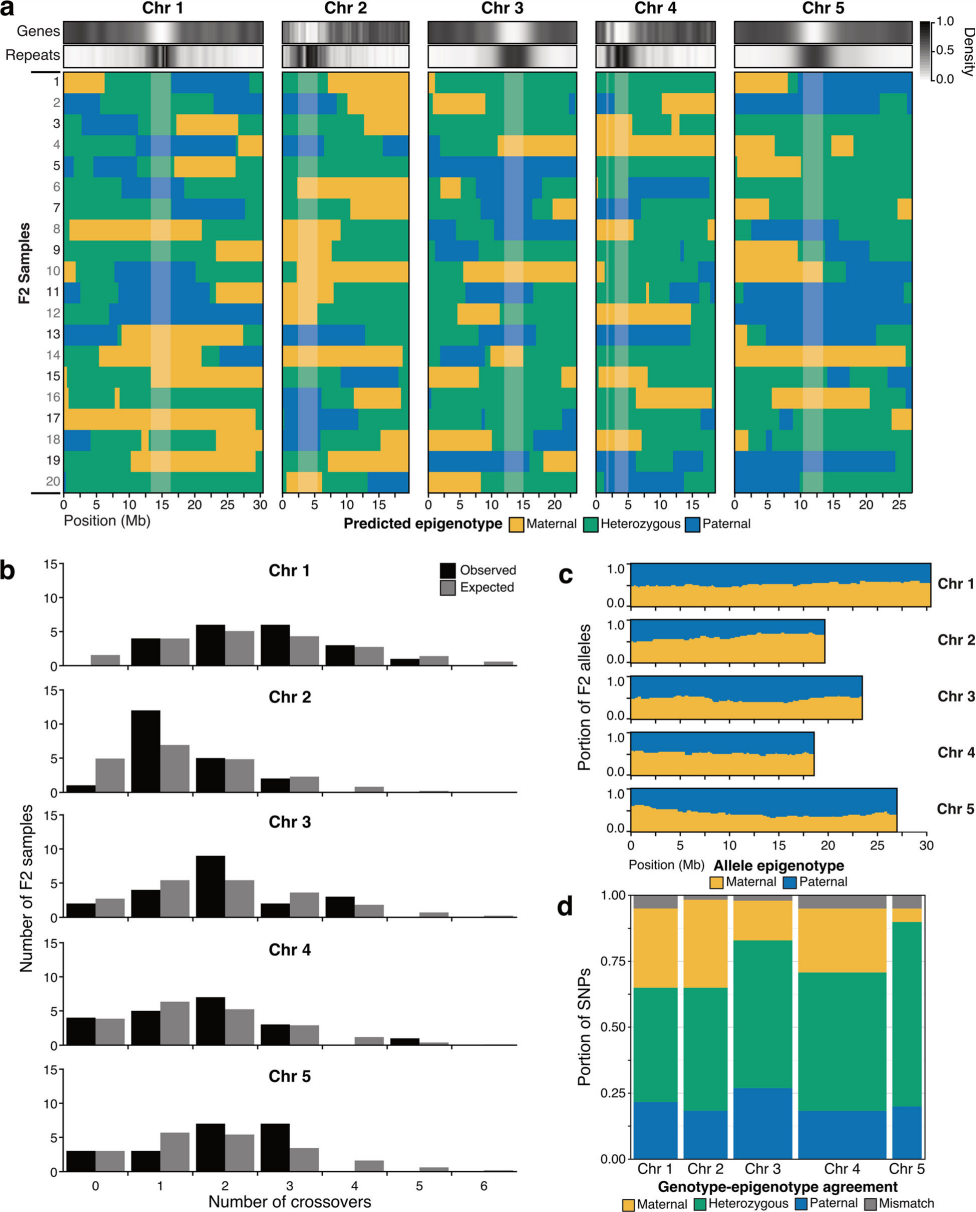
Results of epigenotyping in F2 samples. **a** Final epigenotype maps for all chromosomes in the F2 population of *A. thaliana* Col-0 MA line 49 × line 69. *Yellow* indicates homozygous maternal, *green* heterozygous, and *blue* homozygous paternal. *Lighter color background* denotes the centromere. Heatmaps above chromosomes show gene and repeat density across the chromosome. **b** Distribution of observed number of crossovers per chromosome, in *black*, compared to expected number based on Poisson distribution, in *gray*. **c** Epigenotype predicted allele frequencies across each chromosome. From the law of segregation, maternal (*yellow*) and paternal (*blue*) alleles are expected in a 1:1 ratio. **d** Agreement of predicted epigenotype based on epigenotype map and predicted genotype based on reads for F2 samples at SNPs. *Width of bar* indicates number of SNPs on the chromosome. *Yellow*, *green*, and *blue* indicate both methods predicted maternal homozygous, heterozygous, and paternal homozygous, respectively. *Gray* indicates mismatch between the methods

Confident the epigenotyping procedure worked as intended, the procedure was applied to all samples in the genetic cross using cytosines in all sequence contexts (CG, CHG, and CHH) with 50-kb bins and centromere masking. Methylomes of the G′1 generation and G′2 generation were used as parental replicates. The final maps showed large contiguous blocks of consistent epigenotype with inferred locations of crossovers (Fig. 4a). The average number of crossovers observed in the F2 samples per chromosome compared well to previous studies using genetic approaches [45, 46] (Additional file 1: Table S5). There was no significant difference in the observed distribution and expected distribution of crossovers for each chromosome (Fig. 4b; Additional file 1: Table S5). Additional support for high-quality epigenotype maps was observed by comparing expected and observed allele frequencies in the F2 samples. From the law of segregation, maternal and paternal alleles should occur in equal frequencies at each locus in the F2 samples. Inferring F2 alleles from the epigenotype map, the expected 1:1 ratio could not be rejected for any bin on any chromosome (Fig. 4c; Additional file 1: Table S6).

The parental samples of this cross are nearly genetically identical; however, 36 SNPs were identified between later generations of lines 49 and 69 [40]. After eliminating SNPs that had not occurred in the parents, SNPs that could not be differentiated with bisulfite sequencing, and SNPs with insufficient coverage (Additional file 1: Table S7), 18 SNPs remained available for analysis (Additional file 2: Figure S9a). Overall, the epigenotype and SNP genotype were in agreement for 96.4% of the SNPs (Fig. 4d; Additional file 1: Table S8). Most of the F2 samples (12/20) had all SNPs in agreement and 6/20 samples had only a single mismatch, which could be due to a number of reasons (Additional file 2: Figure S9b). Five of the 13 inconsistencies were centromeric, which was known to be more difficult to epigenotype due to the repeat content. Five other mismatch positions could be due to sequencing errors. For example, a predicted heterozygous SNP occurred in F2-3 on chromosome 1 at 23 Mb in a large block of predicted homozygous maternal. The SNP genotype could be incorrect to a sequencing error on the one read which supports the heterozygous genotype (Additional file 1: Table S8). Using multiple independent populations with and without genetic variation we conclude that we are able to create a genetic map solely using DNA methylation data.

### Inheritance of parental epialleles in F2 progeny

To observe inheritance of DNA methylation states of spontaneous epialleles, 107 DMRs between the parents were identified. If DNA methylation inheritance is mainly additive, a maternal homozygous F2 at a DMR is expected to have a methylation level near the methylation level of the mother. Similarly, a paternal homozygous F2 at the DMR would be expected to have a methylation level near the father. A heterozygous F2 would be expected to have a methylation level between the mother and father levels. Because the parent samples sequenced were siblings of the plants used in the cross, it was important to filter DMRs that were specific to the sequenced sibling and were not due to a spontaneous epiallele. For each line, DMRs were identified between each of the parents, and their subsequent G′1 and G′2 progeny (Additional file 1: Table S9 and Table S10). These regions were eliminated and 100 DMRs remained for subsequent analysis.

**Fig. 5:**
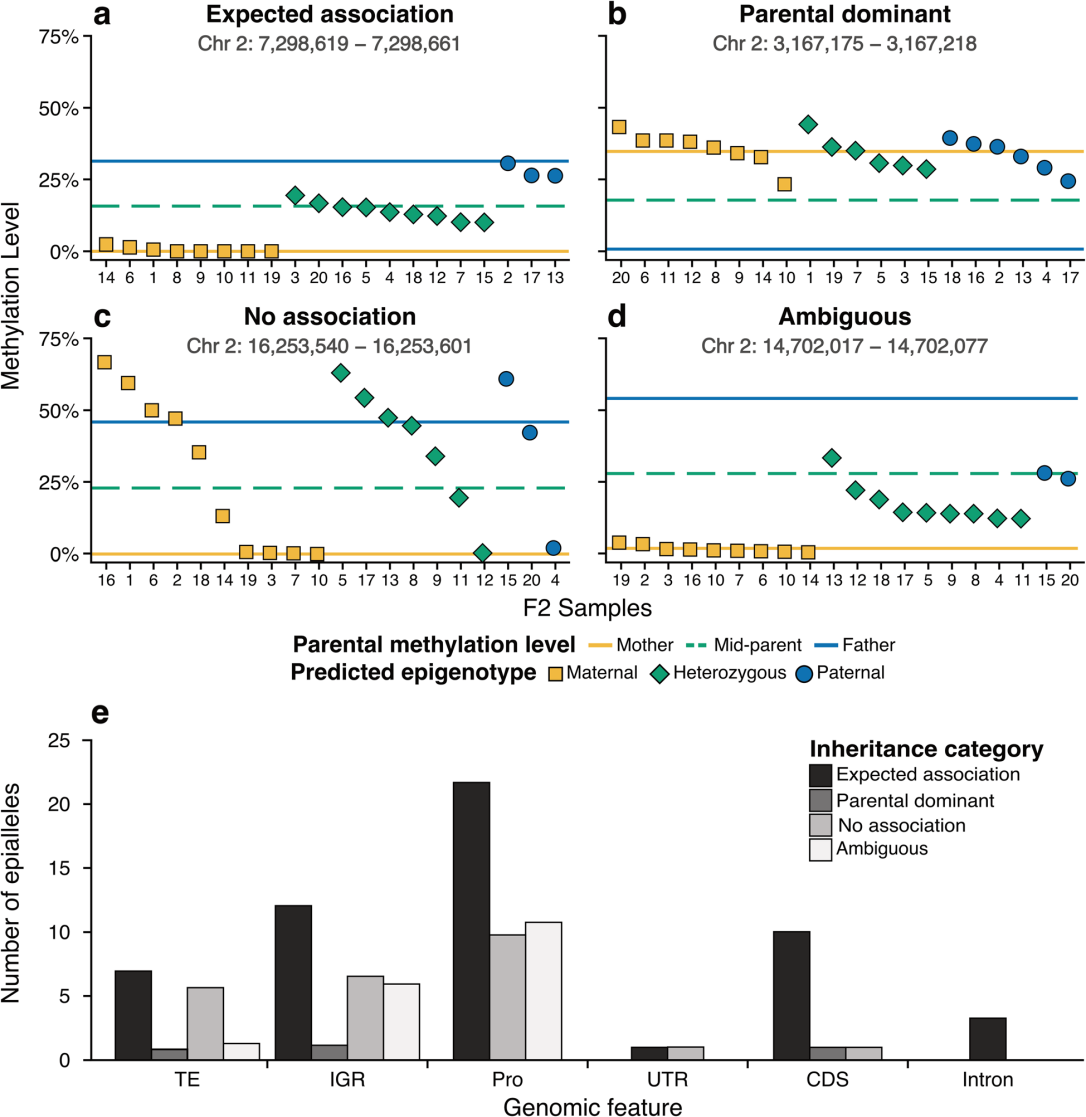
Inheritance patterns of epialleles. Examples of **a** expected, **b** parental dominant, **c** no association, and **d** ambiguous DMRs identified. *Yellow* and *blue lines* indicate methylation level for mother and father, respectively. *Green dashed line* is the computed mid-parent methylation level. *Yellow square*, *green diamond*, and *blue circle* indicate predicted epigenotype of each F2 individual at the DMR as maternal homozygous, heterozygous, and paternal homozygous, respectively. **e** Distribution of underlying genomic characteristics of regions identified by inheritance category. *Abbreviations*: *TE* transposable element, *IGR* intergenic region, *Pro* promoter, *UTR* untranslated region, *CDS* coding sequence

For each region of differential methylation between the parents, the epigenotype for each F2 was determined and the methylation level across the region was computed. Then, the distribution of methylation levels for the F2s with the same epigenotype was compared for differences in mean methylation level between the epigenotypes (Additional file 2: Figure S10; Additional file 1: Table S11). Regions were then categorized into four groups based on the observed pattern of inheritance (Additional file 1: Table S12). In the first group, the distribution of methylation levels agreed with the epigenotype (Fig. 5a). These regions indicated additive methylation inheritance. The second group, parental dominant, consisted of regions where all F2 samples had a methylation level close to one parent, regardless of epigenotype (Fig. 5b). These regions could be explained by trans-chromosomal methylation (TCM) and trans-chromosomal demethylation (TCdM) [31]. The third group of regions showed no association between methylation level and epigenotype (Fig. 5c). These regions could occur as a result of spontaneous events. Regions in the final group were ambiguous, likely a result of the small sample size or large variability of methylation levels of the F2s (Fig. 5d).

Over half of the regions had the inheritance pattern expected by additive methylation inheritance (Additional file 2: Figure S11). In these regions where parental methylation is substantially different, a high proportion of additive inheritance was still observed. Only three regions were categorized as a parental dominant region, suggesting that TCM and TCdM are not commonly associated with spontaneous epialleles. In the cases where small sample size or high variability affected classification, increasing the number of F2 samples analyzed would potentially correct misclassifications.

We were also interested to see if there was any association between inheritance pattern observed and the underlying genetic characteristics (Fig. 5e; Additional file 1: Table S12). Similar to the transgenerational MA lines, most epialleles were in gene promoters. There did not appear to be a correlation between inheritance pattern and genomic feature.

## Discussion

Plants have multiple pathways for maintaining DNA methylation. Although the epimutation rate of individual cytosines is high (10^−4^ per generation per haploid genome) [41], methylation across regions is very stably inherited (Fig. 1b). Of the methylated regions in the genome, less than 0.003% are not faithfully inherited by the next generation. The combined methylation across a region is more crucial than methylation at single positions because it is altered methylation of regions, not positions, that can have documented effects in plants [14–16]. When a spontaneous epiallele arose, we observed approximately 45.3% of the newly formed methylation states are subsequently stably inherited over the generational timescales explored (Fig. 1c). In systems with perturbed methylomes like epiRILs, some regions of abnormal methylation return to the original methylation state after several generations [18, 19, 49]. This suggests there are underlying characteristics leading to the re-methylation, such as sequence, small RNAs, or transposable element content. Most regions, however, are maintained in the newly methylated state. DNA methylation is dynamic but also incredibly stable between generations for most of the methylome.

Variation in DNA methylation is a potential source of alleles that can lead to natural phenotypic diversity. Crop plants tend to have large genomes and many silenced regions, which could provide an even greater source of phenotypic variation as compared to *A. thaliana*. This could be particularly useful for agriculturally important traits. With the invention of epimutagenesis and targeted epigenome editing, it is important that induced epialleles are stable through several generations for them to be useful in crop improvement [50]. The research suggests that although most methylated regions of the genome are stably inherited, locations of natural epialleles have varying stability over generational time. Future work will be needed to explore the factors affecting epiallele stability to avoid inducing epialleles that are unlikely to be stable.

DNA methylation in plants is inherited in *cis*, which has allowed us to develop a procedure to determine genotype from only DNA methylation (Fig. 2). The epigenotyping procedure performed well for simulated data, previously published epiRILs, and a cross between *A. thaliana* accessions (Fig. 3). The procedure was applied to genetically identical F2 progeny and the resulting maps agreed well with the expected number of crossovers in *A. thaliana*, with the expected allele frequencies based on Mendelian inheritance and with SNP data (Fig. 4). This method provides an important first step for epigenetic quantitative trait loci (epiQTL) identification in genetically identical individuals. With additional testing and validation, epigenotype maps created from this procedure can be used to link complex traits to regions of differential methylation in experimental and natural plant populations, including crops.

The epigenotyping procedure has allowed us to uncouple newly formed epialleles from potential genetic causes to accurately identify spontaneous epialleles and more clearly understand patterns of inheritance. Of the epialleles differing between the parents, over half show expected additive inheritance in the F2 offspring (Fig. 5). Few regions showed possible evidence of TCM or TCdM [31]. Sampling additional F2 progeny would be beneficial for future studies to accurately categorize regions with ambiguous patterns.

## Conclusions

This study reveals that DNA methylation within discrete regions is stably inherited across generations with few spontaneous epialleles arising each generation even though the single cytosine epimutation rate is much higher. DNA methylation is inherited in *cis*, which allowed the creation of a procedure to create accurate epigenotype maps in offspring of a genetic cross with almost no genetic variation. Applying this procedure, more than half of differentially methylated regions between the parents were inherited as expected, with DNA methylation levels in the region in agreement with the predicted epigenotype.

## Methods

### Plant material

Seeds from Shaw et al. [38] were obtained for generations indicated in Fig. 1a. Seeds were planted and grown in 16-h day lengths and tissue was harvested from young leaf tissue. Tissue was flash frozen in liquid nitrogen and DNA was isolated using a Qiagen Plant DNeasy kit (Qiagen, Valencia, CA, USA) according to the manufacturer’s protocol.

### MethylC-seq library construction and sequencing

Line 12 samples, *met1-3*, Col-0 wild type, *met1* epiRILs, C24 wild type, Ler wild type, and C24-Ler F2 samples were previously published [31, 33, 39, 41, 47, 48]. Raw sequence reads were downloaded and reanalyzed. The libraries for the C24-Ler F2 samples comprised paired end reads, so to maintain consistency with other samples, only the first reads were used.

All other libraries were prepared according to the protocol described in Urich et al. [51]. MethylC-seq libraries were sequenced to 150 bp using Illumina NextSeq500 (Illumina, San Diego, CA, USA).

### MethylC-seq sequencing analysis

MethylC-seq reads were processed and aligned as described in [52]. Briefly, reads were trimmed for adapters using Cutadapt v1.1.0 [53], parameters minimum quality score 10 and minimum read length 30 and aligned to the TAIR10 reference genome [54] using Bowtie v1.1.0 [55] with parameters “-k 1 –m 1 –chunkmbs 3072 –best –strata –o 4 –e 80 –l 20 –n 0”. Only uniquely mapped reads were retained. Non-conversion rate (the rate which unmethylated cytosines fail to convert to uracil) was calculated from reads aligning to the chloroplast. Positions were considered methylated based on the binomial test followed by Benjamini–Hochberg false discovery rate (FDR) correction. Non-conversion rate was used as the expected probability for the binomial test and only positions with at least three mapped reads were included. Methylation level is computed as the weighted methylation [43]. The weighted methylation level was calculated as mC/(mC + uC) where mC is the number of methylated reads and uC is the number of unmethylated reads.

### Identification of methylated regions

Methylomes generated for transgenerational lines 12 and 69 were computationally combined to form pan-methylomes for lines 12 and 69 independently. Specifically, the number of methylated and total (methylated plus unmethylated) reads was summed at each position across all samples in the line. Additionally, unmethylated pan-methylomes were generated by setting methylated reads to zero, while maintaining the total number of reads as reported in each line’s pan-methylome. For each line, the methylpy DMR identification program [52] was applied, comparing the pan-methylome and unmethylated pan-methylome to identify all C DMRs, i.e., CNN DMRs (N = A, C, G, T). Parameters used were 3000 simulations, 100 significant tests, and 250 bp maximum DMR distance. Of these regions, regions 40+ bp long that had at least ten cytosines covered by at least three reads in the combined methylome were retained. A one-sided z-test was used to test for expected methylation level of 25% in at least one generation, i.e., 25%/8 generations = 3.125% for line 12 and 25%/10 generations = 2.5% for line 69. The resulting *P* values were adjusted using Benjamini–Hochberg correction (n = 33,208 for line 12 and n = 31,569 for line 69). After computing methylation level for each generation in each line, regions where all generations of a line had a methylation level less than 10% were removed. The mean length for methylated regions was 1138 and 1319 bp for lines 12 and 69, respectively.

### Identification of transgenerational epialleles

DMRs were identified for all samples within a line using methylpy as described above. Of these regions, only regions 40+ bp long that had at least ten cytosines covered by at least three reads in all generations of a line and at least 20% difference in methylation level between the highest and lowest methylation level of generations in a line were retained. For each region in a line, a one-sided z-test was performed to test for a significance greater than 25% difference in methylation level between adjacent generations. Resulting *P* values were adjusted using Benjamini–Hochberg correction (n = 910 for line 12 and n = 1674 for line 69). A region is considered an epiallele between generations with an adjusted *P* value ≤0.05 (Additional file 1: Tables S13 and S14). Regions that did not overlap with identified methylated regions were eliminated. Regions with at least one epiallele were considered an epilocus. The mean length of epialleles in both lines was 94 bp. Due to the unequal distribution in length between identified epialleles and methylated regions, estimates of stability were computed for regions of 100 bp.

### Heatmap construction

Methylated regions and epialleles identified in lines 12 and 69 were merged. At imperfect overlaps, the minimum start and maximum end was used to create the merged region. Methylated regions, excluding identified epialleles, were ordered using R’s ward.D clustering of the Euclidean distance of sample methylation values between regions [56]. Line 12 epialleles were ordered by R’s ward.D clustering of the Euclidean distance of methylation values for line 69 samples. Line 69 epialleles were similarly ordered using methylation values of line 12 samples. Overlapping epialleles were ordered using methylation values of samples in both lines.

### Categorization of epialleles by genomic features

A genomic feature map was created such that each base pair of the TAIR10 genome [54] was assigned a single feature type (transposable element, promoter, untranslated region, coding sequence, intron, and non-coding RNA) based on the TAIR10 annotation [54]. Promoters were defined as 1 kb upstream of the transcription start site of protein-coding genes. At positions where multiple feature types could be applicable, such as a transposon in an intron or promoter overlapping with adjacent gene, priority was given to non-coding RNA (highest), untranslated regions, introns, coding sequences, promoter, and transposon (lowest). Positions without an assignment were considered intergenic. Genomic feature content of each epiallele and methylated region was assigned proportionally based on the number of bases in each category.

### Experimental set-up for genetic cross

An individual from line 49 generation 23 (line 49-G23) and an individual from line 69 generation 19 (line 69-G19) of Shaw et al. [38] were each self-fertilized and grown. Individuals from the offspring were crossed such that line 49-G24 was the mother of the cross and line 69-G20 was the father. Siblings of these individuals were used for WGBS. Offspring of the cross were grown to create the F1 generation. A single randomly selected individual from the F1 was self-crossed and offspring, the F2 generation, were grown. Twenty individuals from the F2 generation were randomly selected for WGBS and subsequent analysis.

Additionally, the individuals from line 49-G20 and line 69-G24 used for the cross were self-crossed and created lines 49-G′1 and 69-G′1, respectively. One individual from those populations was randomly selected for WGBS and was self-crossed again to create lines 49-G′2 and 69-G′2. A single individual from each population was randomly chosen for WGBS.

Plants were grown in 16-h day lengths and tissue was harvested from young leaf tissue. Tissue was flash frozen in liquid nitrogen and DNA was isolated using a Qiagen Plant DNeasy kit (Qiagen, Valencia, CA, USA) according to the manufacturer’s protocol.

### Feature density and definition of centromere

Density as number of genic base pairs per 100 kb was computed for each chromosome. A genic base pair is one that occurred within the gene feature coordinates of the TAIR10 [54] annotation. A spline was constructed using R’s smooth.spline function [56] and the minimum of that spline was considered the centromere center for the chromosome. For chromosomes 1, 2, 3, and 5, the centromere was defined as 1.5 Mb on either side of the center. This was 13.3–16.3 Mb for chromosome 1, 2.4–5.4 Mb for chromosome 2, 12–15 Mb for chromosome 3, and 10.4–13.4 Mb for chromosome 5. Due to the bimodal gene distribution caused by a heterochromatic knob on chromosome 4, the centromere was defined as 1.6–1.9 Mb and 2.9–5 Mb.

### Epigenotyping procedure by MethylC-seq

First, differentially methylated positions in all sequence contexts were identified between the mother and father. A position was considered differentially methylated if coverage was at least three in both parents and only one parent was methylated based on the binomial test. Using these positions, the weighted methylation [43] was computed for all samples (mother, father, F2s, G′1 s, and G′2 s) keeping only positions with at least three reads in all samples.

Next, each chromosome was analyzed independently. The chromosome was broken into bins of size *x*. Then bins with less than three positions were combined with a neighboring bin. For each bin, feature vectors (methylation level at all positions within the bin) were created for the mother sample and father sample. These feature vectors were combined to create a mid-parent value (MPV) feature vector as the average methylation between the parents at each position. The G′1 and G′2 samples were used as parental replicates and additional MPV feature vectors were created using the G′1 and G′2 samples as the corresponding MPV replicates. The maternal, paternal, and MPV feature vectors were used to train a logistic regression classifier using the sklearn toolkit v0.17.1 [57] in python v3.5.2. The classifier was run with one-versus-rest multiclass option and the liblinear solver. Classification states were weighted 0.25 for mother and father and 0.5 for MPV since F2s are expected to follow a 1:2:1 ratio. This trained logistic regression classifier was applied to the bin in all samples, including the mother, father, and MPV samples. The predicted probability for each state (maternal, paternal, MPV) was determined and the state with the highest probability at the bin was reported as the preliminary predicted epigenotype.

Then, for each chromosome a transition matrix was computed using all samples except the mother and father. The transition from class *l* to class *k* is the sum of bins *i*, *i* =1 to N where bin *i* is class *l* and bin *i* +1 is class *k*. A pseudo-count of 1 was included for each transition, and the transition matrix was normalized.

A hidden Markov model was constructed with three states (mother, mid-parent, and father) for each bin and the transition matrix as transition probabilities. For all states of each bin, the logistic regression prediction probabilities were used as the emission probabilities. Then the forward-backward procedure [44] was applied to each chromosome of each sample. The forward-backward procedure identifies the most likely state at each bin based on the posterior probability distribution. Centromere regions were masked such that only transition probabilities contributed to the posterior probability, i.e., emission probabilities were 1.0. The state with the highest probability for each bin was reported as the forward-backward prediction and the posterior probabilities were used as forward-backward prediction probabilities.

Next, a new transition matrix was computed using the forward-backward predictions.

The Viterbi algorithm [44] was applied using forward-backward prediction probabilities as the emission probabilities and new transition matrix as the transition probabilities. It finds the most likely sequence of classes using maximum likelihood. Again, in centromeric regions, only the transition probability was used. The traceback procedure of the Viterbi decoding algorithm was used to assign the final class prediction or predicted epigenotype.

### Simulation testing

Simulations to test the epigenotyping procedure used the observed methylation values of differentially methylated positions on chromosome 3 for parents of the line 49–line 69 cross. Twenty samples were created with zero to 19 possible breakpoints equally spaced along the chromosome. For all samples, genotype (maternal, heterozygous, paternal) was randomly assigned to each region between breakpoints along the chromosome. Genotype probabilities were 0.25, 0.5, 0.25 for maternal, heterozygous, and paternal to emulate the expected probability of the F2s. Adjacent regions could be assigned the same genotype; thus, a sample with, for example, five possible breakpoints could have zero to five actual breakpoints.

Let *x* be the expected methylation level of the assigned genotype which equals the maternal, paternal, and mid-parent methylation level at a position. Let *y* be the error parameter such that the assigned methylation level at a position is randomly chosen between min (0, *x* – *y*) and max (*x* + *y*, 1). Simulations were run for values of *y* from 0 to 1 in 0.1 increments. The bin size parameter can have dramatic effects on the results, so the algorithm was run for bin sizes 10, 20, 50, 100, 200, and 500 kb. The epigenotyping procedure was run at each bin size using the observed mother and father samples as the parental samples and 20 simulated samples for a given variability *y*. Centromeric regions were not specified.

Accuracy of the prediction made by the algorithm was computed as the F_1_-score using sklearn toolkit [57] with “micro” for the average parameter using the assigned genotypes as truth. The process of assigning sample genotypes and subsequent analysis was repeated for a total of 25 iterations. The final accuracy reported for each sample, variability, and bin size is the average accuracy of these 25 iterations.

### Epigenotyping *met1* epiRIL lines

The procedure was applied such that the logistic regression classifier was trained to classify WT, heterozygous, and *met1* using the WT sample for the mother and the *met1-3* sample for the father. Individuals of F8 epiRILs are expected to have much lower levels of heterozygosity compared to F2 individuals (0.5 vs 0.0078 for F2 and F8, respectively). To account for this, classification states were weighted by 0.4961, 0.0078, and 0.4961 (127:2:127) for mother, MPV, and father, respectively, based on the expected heterozygosity for F8 individuals. There was strong bias towards the mother (WT) for the logistic regression classifier. The prediction probability for the father (*met1*) was split between the father state and MPV state. The MPV state was not predicted in any sample, including the MPV sample, so the computed transition matrix remained unaffected; however, the forward-backward algorithm overrepresented the mother state due to the biased emission probabilities. To correct this, the emission probabilities used by the forward-backward algorithm were adjusted such that the emission probability of the MPV state was added to the probability of the father state. Due to remethylation events that can occur in epiRILs, only CG positions within the coding regions of gene body methylated genes [48] that were differentially methylated between WT and *met1* were used. The epigenotyping procedure was run using 50-kb bins and centromeres were not specified.

### Epigenotyping C24-Ler F2 samples

The epigenotyping procedure was run using cytosines in all sequence contexts and 50-kb bins. Centromeres were defined as previously described. The genetic maps from Greaves et al. [33] were created using 10-kb bins, so each 50-kb bin from the epigenotyping procedure was separated into five 10-kb bins. Most recombination events occurred in regions where genotype could not be determined in the genetic map. When determining the distance between breakpoints predicted by both maps, if the predicted breakpoint from the epigenotyping procedure occurred within the undetermined genotype region, distance was considered zero. When the predicted breakpoint was outside the undetermined genotype region, distance was calculated from the closest edge of the region to the breakpoint. Bins with unknown genotype were not included when computing agreement between the genetic map and epigenotype map.

### Identification of breakpoints and expected crossover number

For all F2 individuals, breakpoints/crossovers were identified along each chromosome where adjacent bins had different epigenotypes. For each chromosome, a Poisson distribution was fitted using the mean number of crossovers. Confidence interval was identified using sample standard error. Expected number of crossovers per chromosome was found given a Poisson distribution with the mean observed for each chromosome. Probabilities were computed for x = 0 – 6 because fewer than one of 20 individuals were expected to be observed with more than six crossovers. In the F2s, no more than five crossovers were observed. An exact multinomial test [58] was used to test for a difference between expected and observed crossover number for each chromosome. Resulting *P* values were adjusted with Benjamini–Hochberg correction.

### Allele frequency inferred from epigenotype

Allele frequency or genotype ratio was tested using epigenotype predictions of chromosomes 1–5 of F2s from 50-kb bin size. Allele frequency is expected to be 1:1 for maternal and paternal. At each bin, the frequency of each allele was computed and a Chi-squared goodness of fit test was run in R. Resulting *P* values from the chi-squared test were adjusted using Benjamini–Hochberg correction.

### WGBS SNP verification

Based on Ossowski et al. [40], 36 SNPs exist between line 49 generation 31 and line 69 generation 31. A subset of these SNPs were expected to have occurred by generation 24 in line 49 and generation 20 in line 69. Additionally, SNPs of unmethylated cytosine to thymine cannot be used because they are not differentiable in WGBS. Samtools v1.12 mpileup [59] was run on the mapped WGBS reads at positions of all possible SNPs to get read coverage at each SNP. Of the original 36 SNPs, 18 were differentiable between the parent samples, i.e., at least one nucleotide was unique to each sample and had sufficient coverage to predict genotype in all F2 samples. Using the unique nucleotides at each SNP, the genotype of F2s was assigned maternal/paternal if it only had reads matching the unique maternal/paternal nucleotide and assigned heterozygous if it had at least one read matching both.

### Identification of sibling-specific DMRs in parents

DMRs were identified with methylpy in the CNN context for line 49 (mother, 49-G′1, and 49-G′2) using the same parameters as transgenerational DMRs. Resulting DMRs were filtered by length and difference is weighted methylation between the most and least methylated samples such that only DMRs of at least 40 bp and 25% absolute difference in methylation level were retained. For each remaining DMR, a z-test was performed to calculate the *P* value for a greater than 25% difference in methylation level pairwise for line 49-G24 (mother) to 49-G′1 and line 49-G24 (mother) to 49-G′2. Resulting *P* values were corrected with the Benjamini–Hochberg procedure. A DMR was considered an epiallele if at least one comparison was significant, adjusted *P* value ≤0.05. The same procedure was applied for line 69 with line69-G20 (father), 69-G′1, and 69-G′2.

### Identification of DMRs between parents

Using WGBS from line 49-G20 and line 69-G20, the parents of the cross, DMRs in the CNN, or all Cs, context were identified using the same program and parameters as transgenerational DMRs. Resulting DMRs were filtered by length and difference in weighted methylation such that only DMRs of at least 40 bp and 25% absolute difference in methylation level were retained. Regions that overlapped with sibling-specific DMRs were eliminated.

### Categorization of epiallele inheritance patterns in F2 samples

At each identified epiallele differing between the parents, methylation level was computed for the parents and F2s. The F2 epigenotype at each epiallele was assigned from the epigenotype map.

Regions were grouped into four categories: expected association, parental dominant, no association, and ambiguous. To assign each region a category, the Games–Howell post-hoc method [60] was used to compare the difference in mean methylation level for each F2 epigenotype group. For each epiallele, a t-value was obtained for each pairwise comparison between F2 epigenotype groups (maternal, heterozygous, paternal). Regions where a t-value could not be obtained were removed from analysis. Then, 2000 bootstrapped samples were run randomly, assigning the same distribution of epigenotype as the epigenotypes of F2 samples at the epiallele and t-values obtained. This provided a null distribution on t-values to test the observed t-value against. Each comparison was considered significantly different if the observed t-value was greater or equal to the 99th percentile of the bootstrapped t-values.

If all comparisons (maternal–paternal, heterozygous–paternal, heterozygous–maternal) were significantly different, the region was assigned “expected association” as each epigenotype group had a unique mean methylation level. If the average methylation level of all F2 samples was within 10% of one parent’s methylation level and the methylation level of each F2 sample was closer to the same parent’s methylation level, the DMR was assigned “parental dominant”. At regions with no significant heterozygous comparison, there was no association between epigenotype and methylation level and region was assigned “no association”. If only one heterozygous comparison (heterozygous–paternal or heterozygous–maternal) was significant, indicating heterozygous samples had methylation levels similar to one homozygous parental epigenotype, the region was assigned “ambiguous”.

## Additional files

Additional file 1: Supplementary **Tables S1–S14.** (XLS 827 kb)

Additional file 2: Supplementary **Figures S1–S11.** (PDF 15370 kb)
