# Supplementary material for: Stable inheritance of DNA methylation allows creation of epigenotype maps and the study of epiallele inheritance patterns in the absence of genetic variation
Source: Genome Biol. 2017 Aug 16;18:155. doi: 10.1186/s13059-017-1288-x (PMC5559844; doi:10.1186/s13059-017-1288-x)
Supplement: Supplementary file 2 — Supplementary Figures S1–S11. (PDF 15370 kb) [file 13059_2017_1288_MOESM2_ESM.pdf]

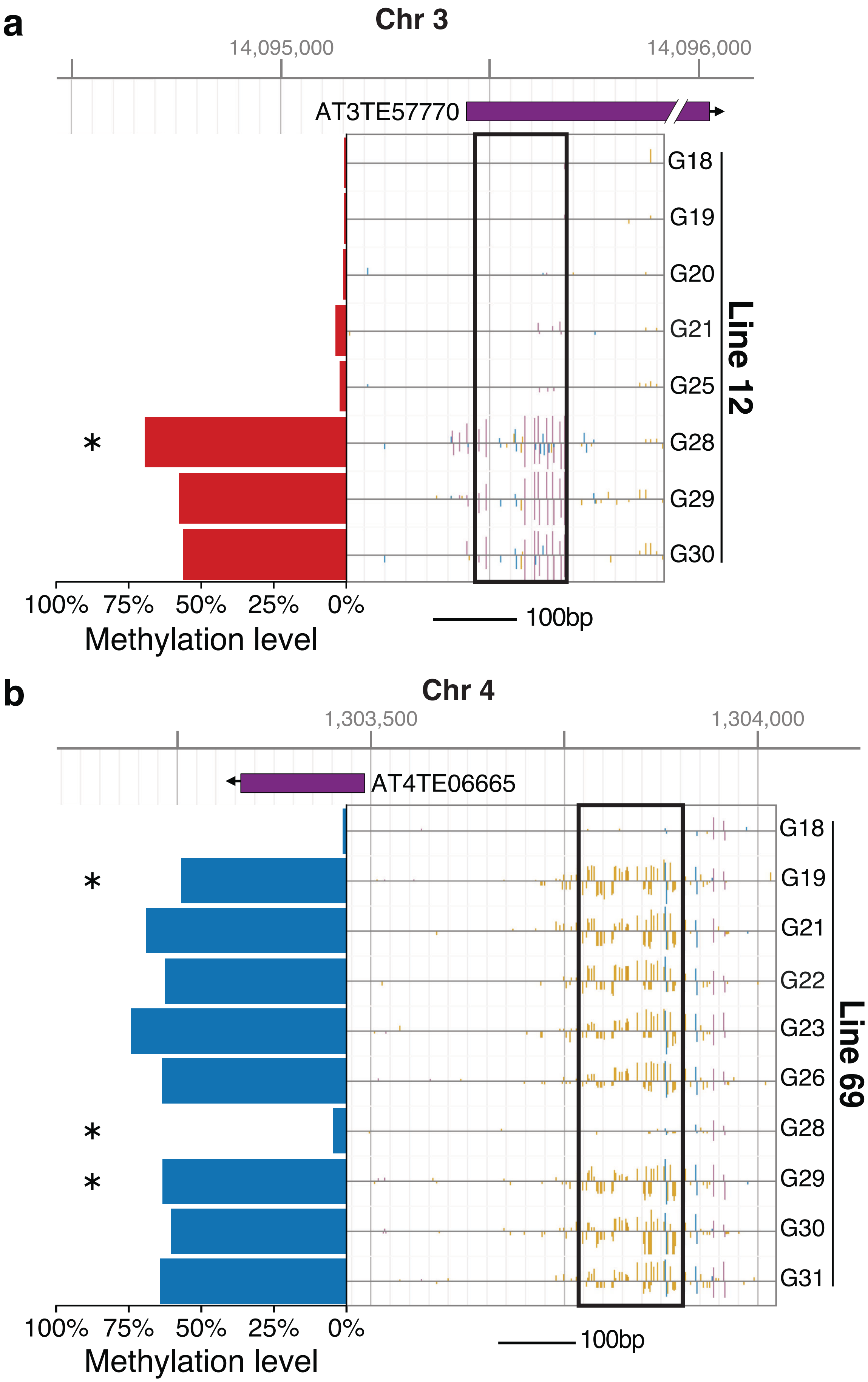

Fig. S1. Examples of transgenerational epiallele stability. Representative epiloci for line 12 (a) with a single spontaneous epiallele and line 69 (b) with multiple spontaneous epialleles. In genome browser view, CG is red, CHG is blue, CHH is yellow and height of the vertical bar indicates methylation level at the cytosine. Bar plot shows methylation level of epilocus for each sample. Epilocus is boxed and star indicates a spontaneous epiallele.

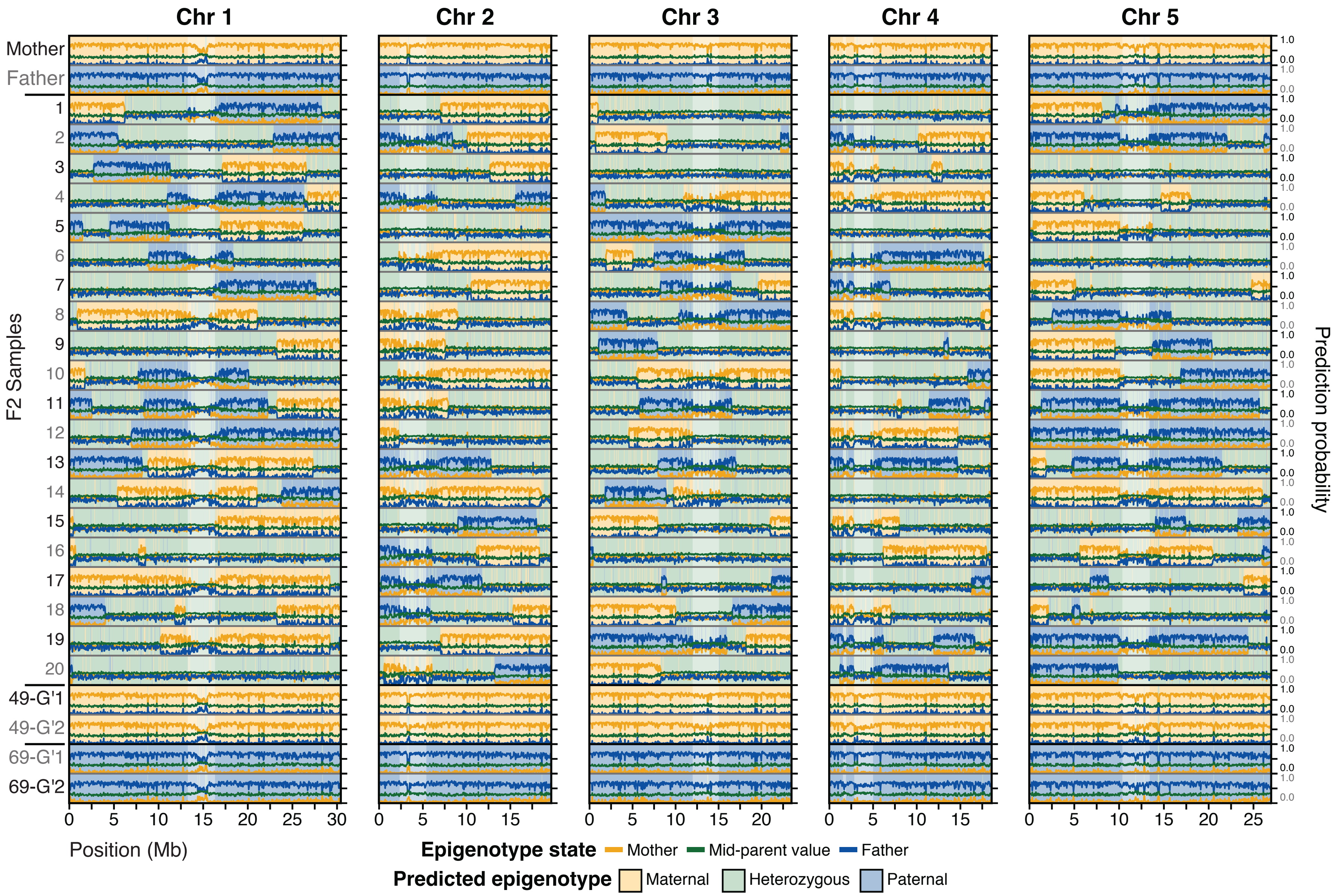

Fig. S2. Results of logistic regression classification in epigenotyping procedure. Results of logistic regression classification step for the mother, father, F2, G′1, and G′2 samples of line 49 x line 69. Procedure was run using 50 kb bins and cytosines in all sequence contexts. Each row represents a sample. Colored lines indicate the prediction probability for each classification from the logistic classifier, yellow for mother, green for mid-parent, and blue for father. Fill color indicates epigenotype prediction, yellow for maternal homozygous, green for heterozygous, and blue for paternal homozygous. Lighter background color denotes centromere.

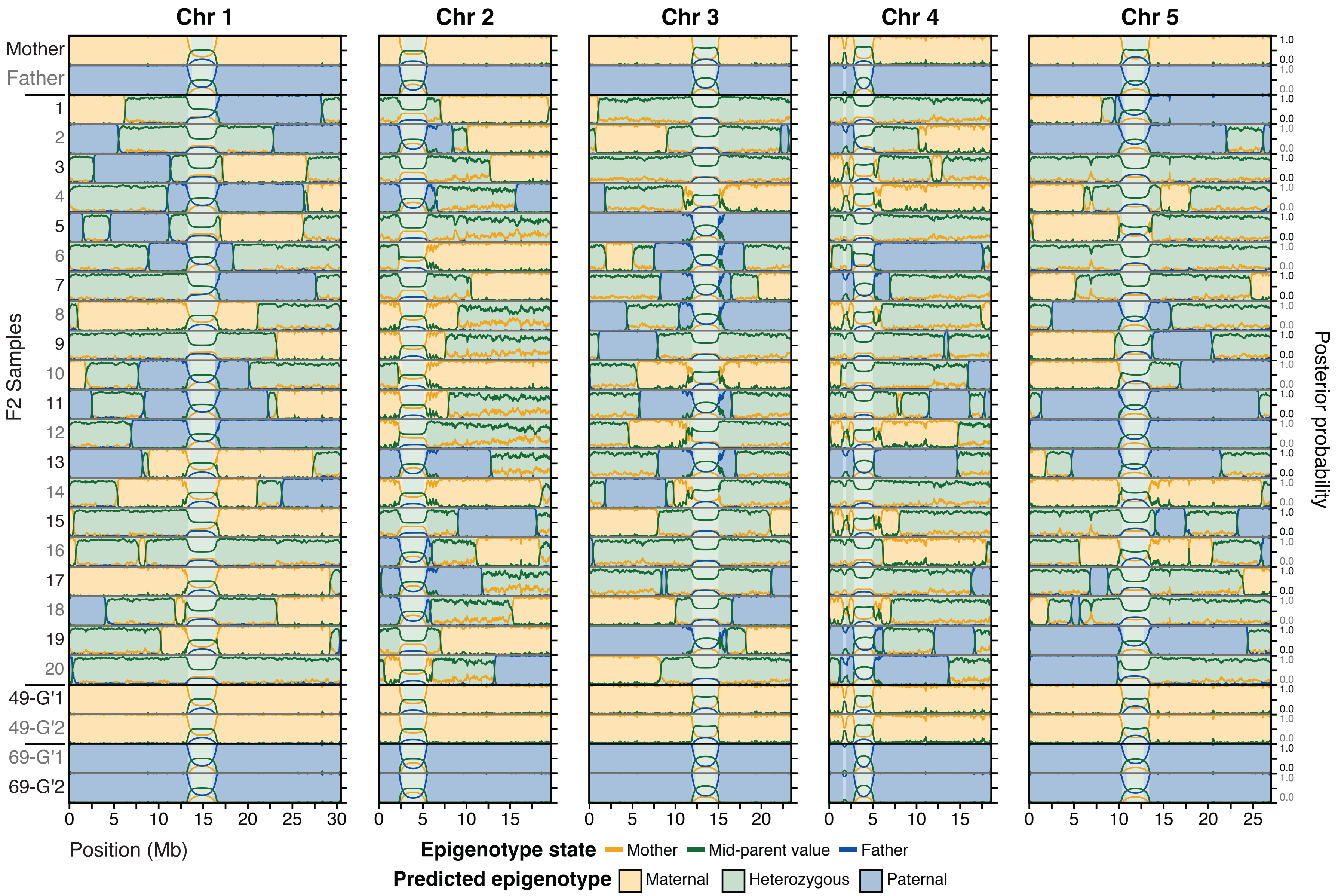

Fig. S3. Results of forward-backward algorithm in epigenotyping procedure. Results of the forward-backward algorithm step for the mother, father, F2, G′1, and G′2 samples of line 49 x line 69. Procedure was run using 50 kb bins and cytosines in all sequence contexts. Each row represents a sample. Colored lines indicate calculated posterior probability of each classification, yellow for mother, green for mid-parent, and blue for father. Fill color indicates epigenotype prediction, yellow for maternal homozygous, green for heterozygous, and blue for paternal homozygous. Lighter background color denotes centromere.

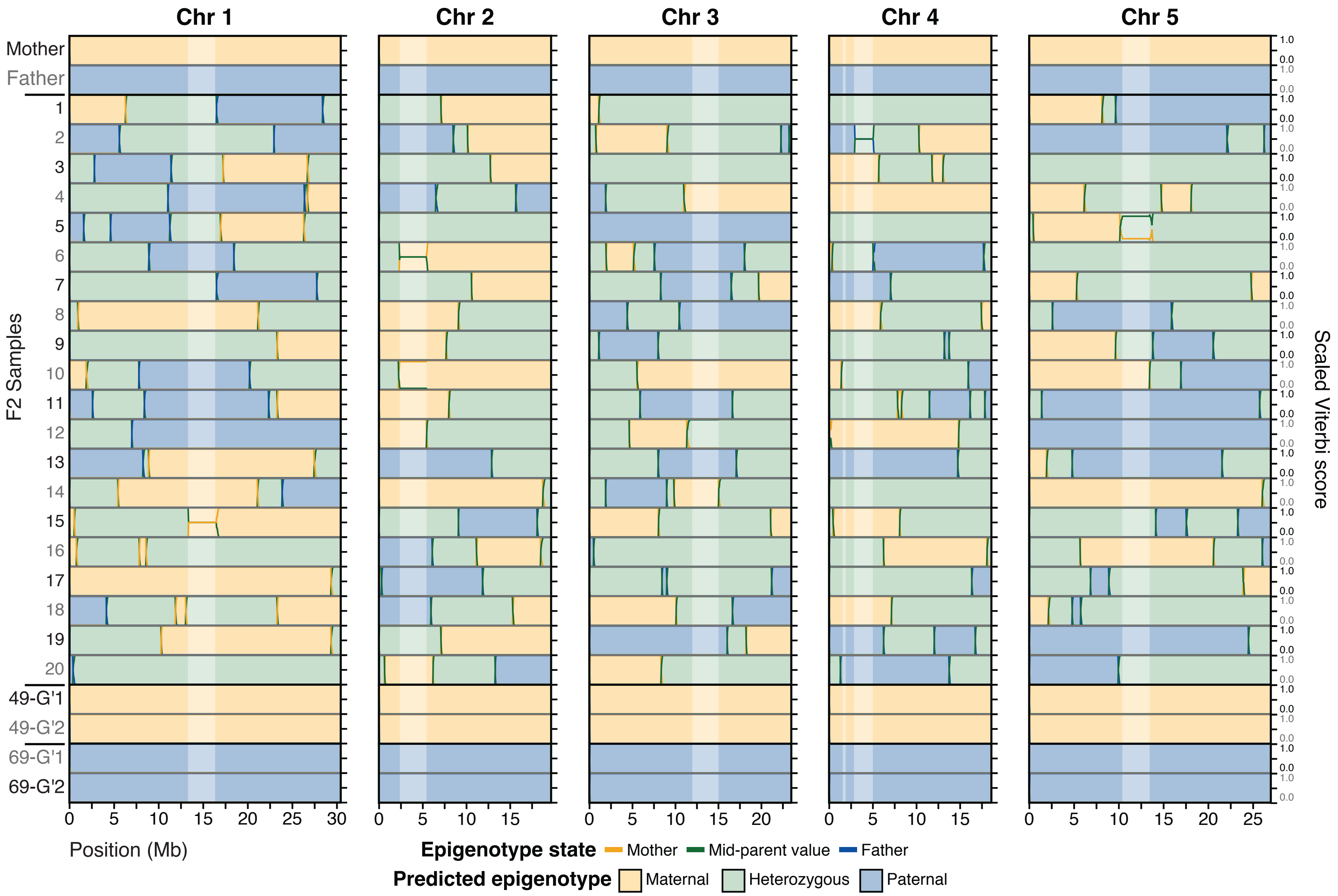

Fig. S4. Results of the Viterbi algorithm in epigenotyping procedure. Results of the Viterbi algorithm step for the mother, father, F2, G′1, and G′2 samples of line 49 x line 69. Procedure was run using 50 kb bins and cytosines in all sequence contexts. Each row represents a sample. Colored lines indicate scaled Viterbi score of each classification, yellow for mother, green for mid-parent, and blue for father. Fill color indicates epigenotype prediction, yellow for maternal homozygous, green for heterozygous, and blue for paternal homozygous. Lighter background color denotes centromere.

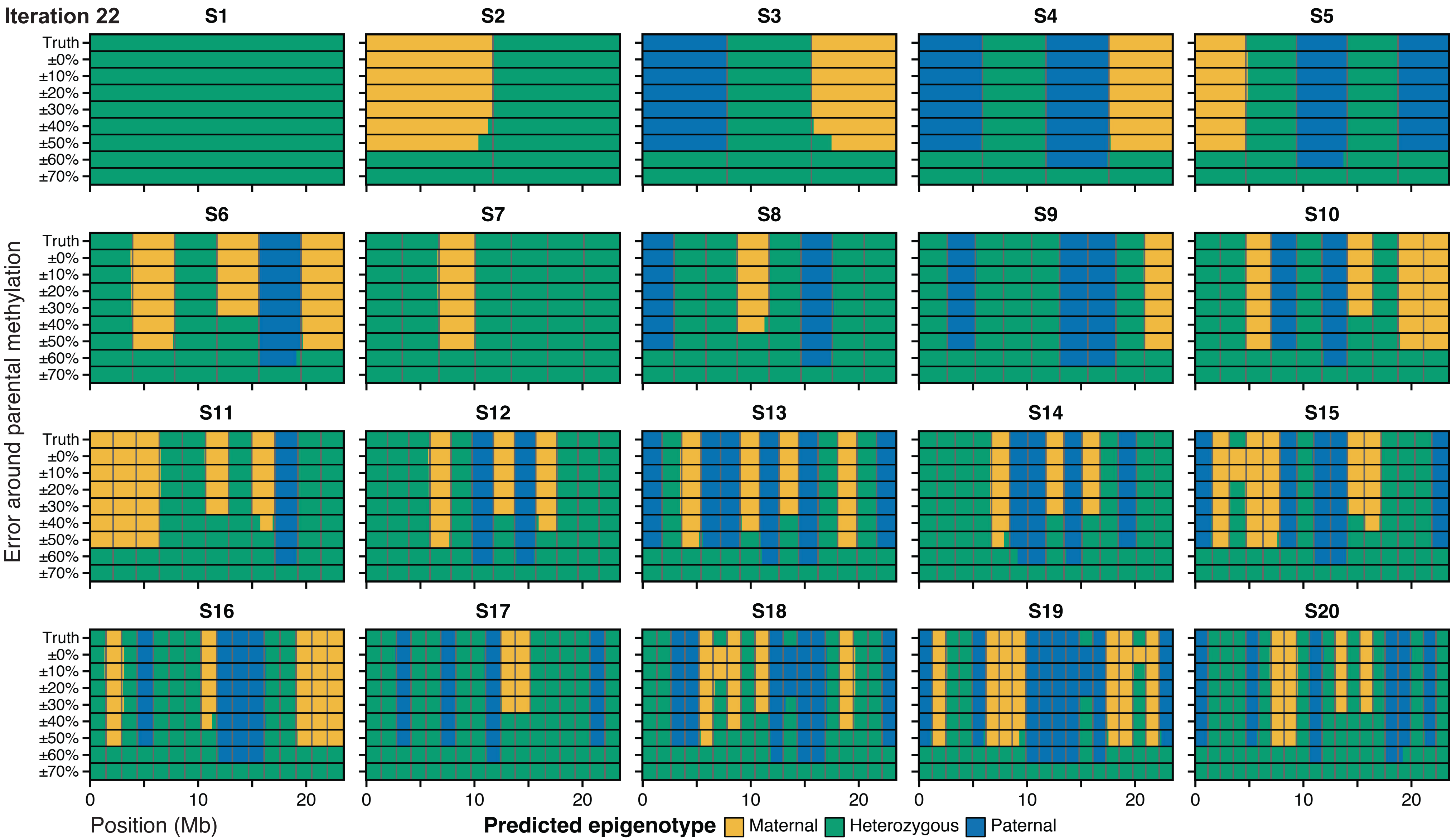

Fig. S5. Example of simulated data. Results of simulated data (see Methods) for simulation iteration 22 with 50 kb bins. For each sample with increasing number of possible breakpoints, the top row indicates the assigned epigenotype with subsequent rows indicating the predicted epigenotype with increasing error value around the expected parental methylation in the simulated methylomes. Gray vertical bars indicate possible breakpoints. Results for error values ±80% - 100% are exclude as they are the same for ±70%.

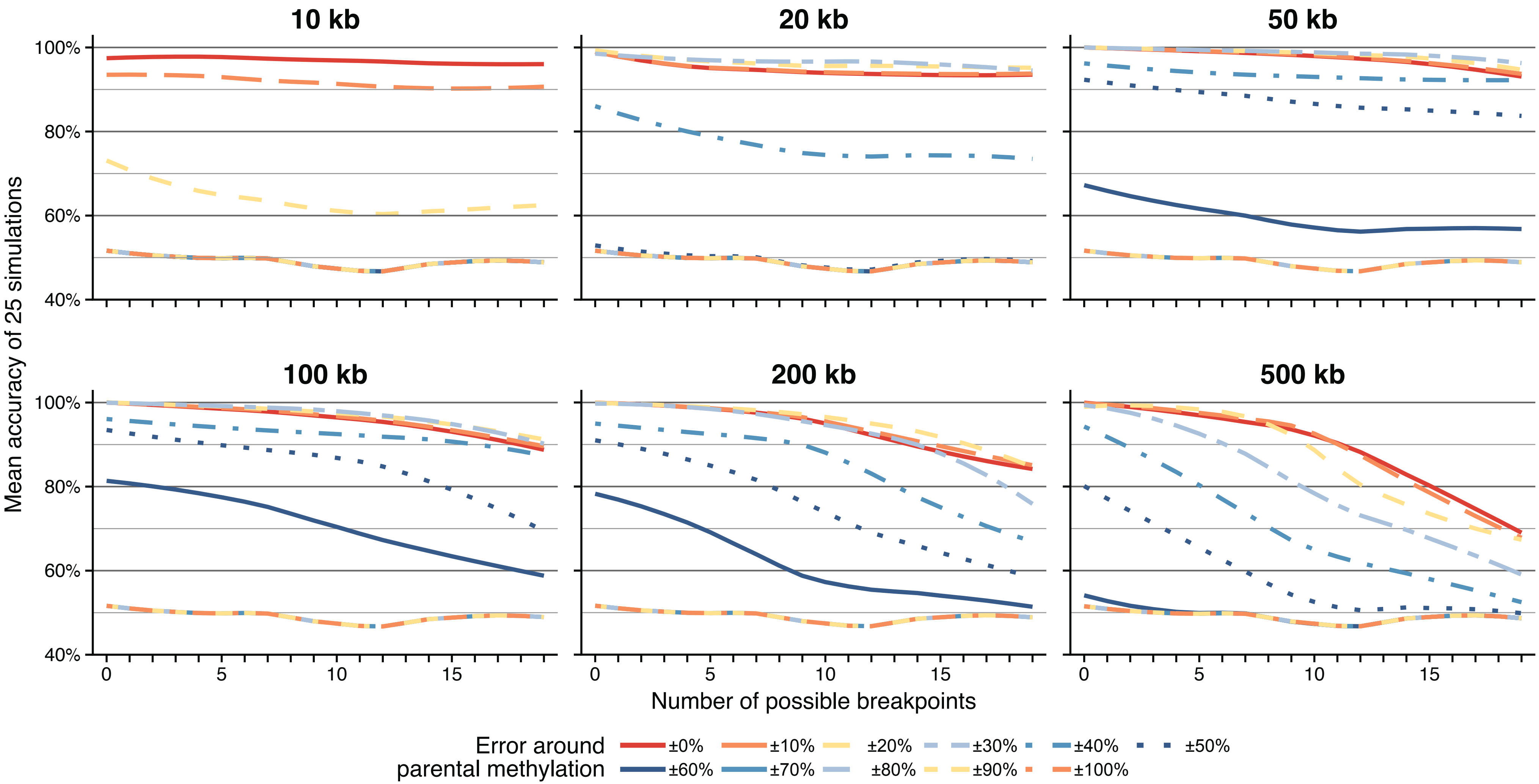

Fig. S6. Overview of accuracy for simulations. Each line is the average accuracy of 25 simulations for each combination of number of possible breakpoints, error around parental methylation, and bin size used. Lines were smoothed using local regression.

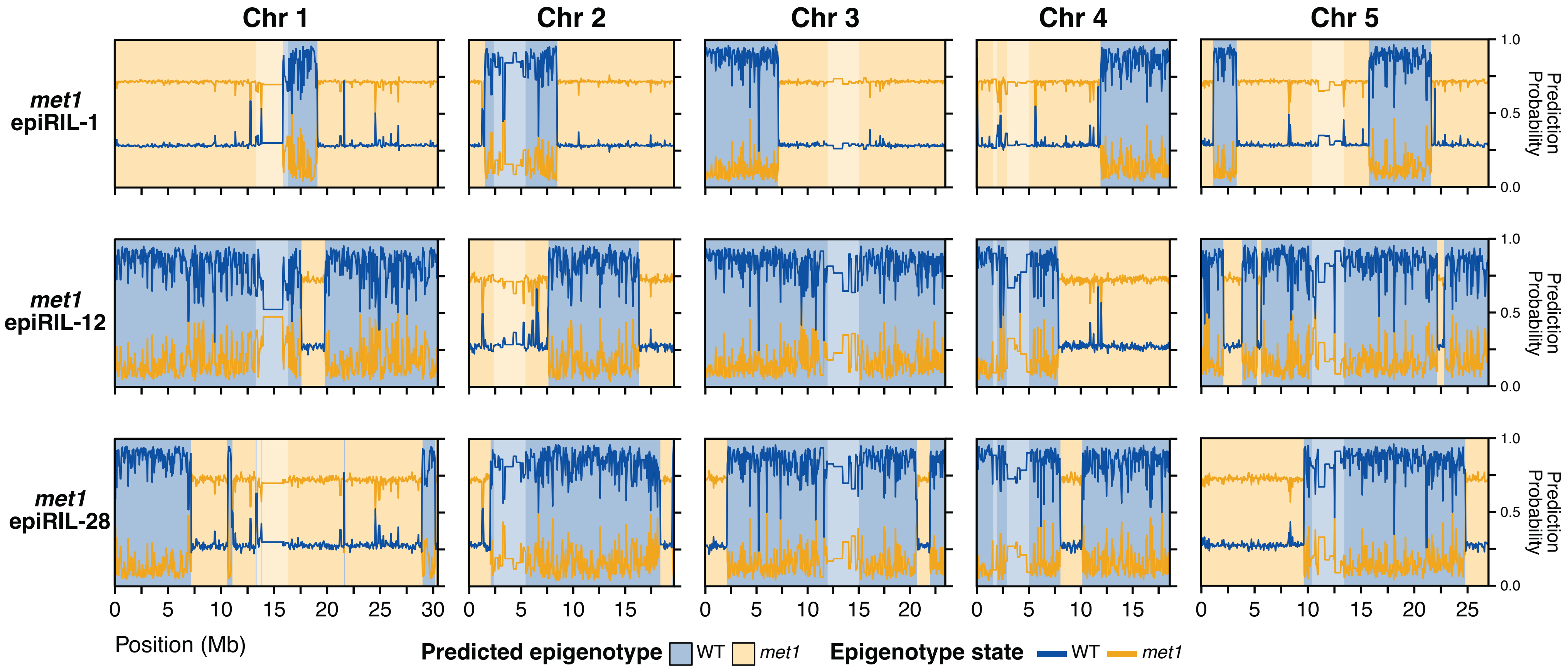

Fig S7. Epigenotype maps for *met1* epiRILs. Results of epigenotyping procedure for three *met1* epiRIL lines. Procedure was run using only CG cytosines within gene body methylated genes with 50 kb bins. Fill indicates epigenotype prediction. Blue denotes WT-derived region of chromosome and yellow denotes *met1*-derived region. Centromere is denoted by lighter fill color. Line indicates prediction probability for each state, WT in blue and *met1* in yellow, based on the logistic regression classifier.

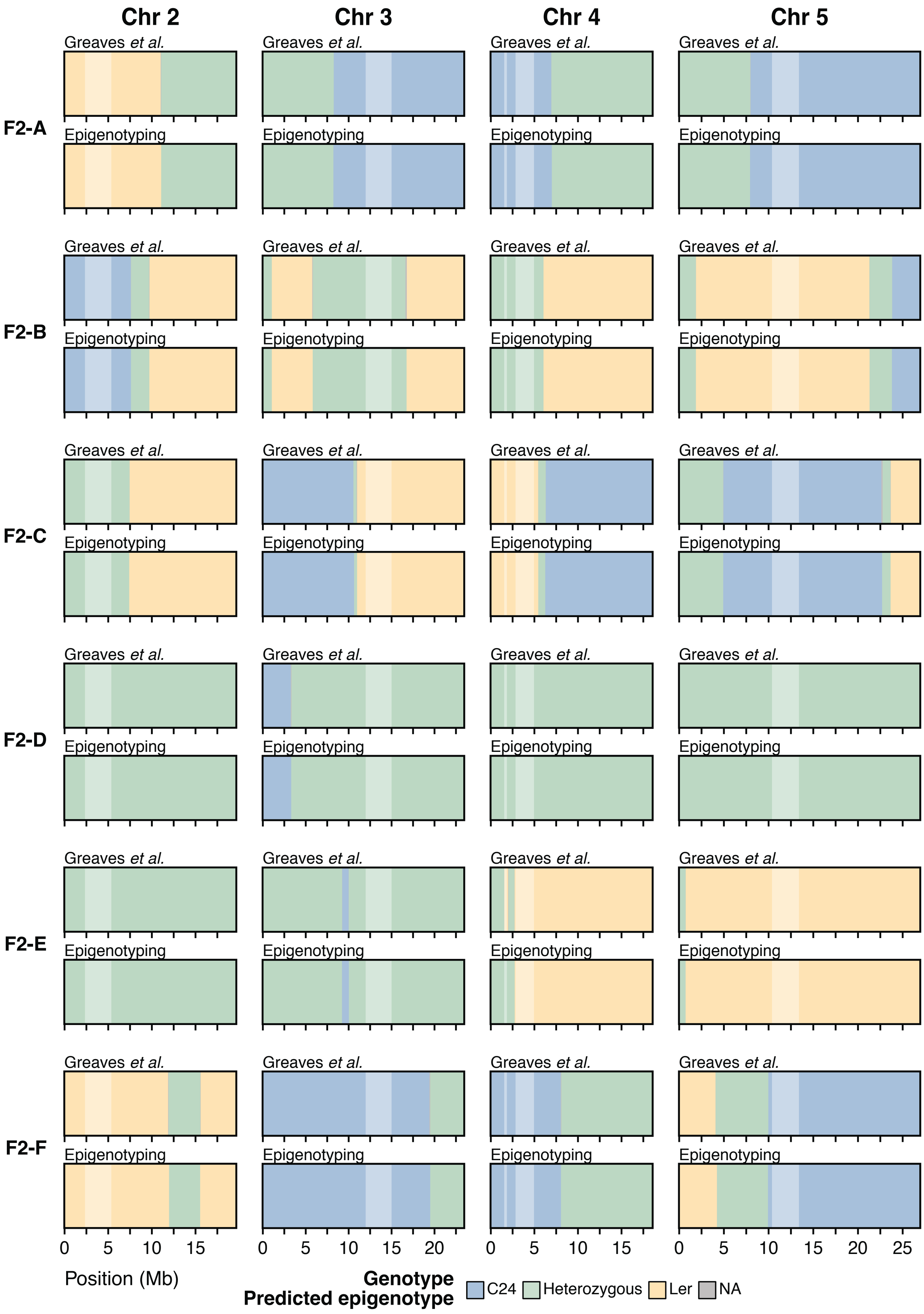

Figure S8. Comparison of SNP-based genetic maps and epigenotype maps for C24-Ler cross. For each chromosome and sample, top plot is the genetic map by Greaves *et al.* (2016). Bottom plot is epigenotype map using cytosines in all contexts with 50 kb bins. Yellow fill denotes maternal homozygous, green denotes heterozygous, and blue denotes paternal homozygous genotype and epigenotype. Gray indicates regions where genotype could not be determined using SNPs.

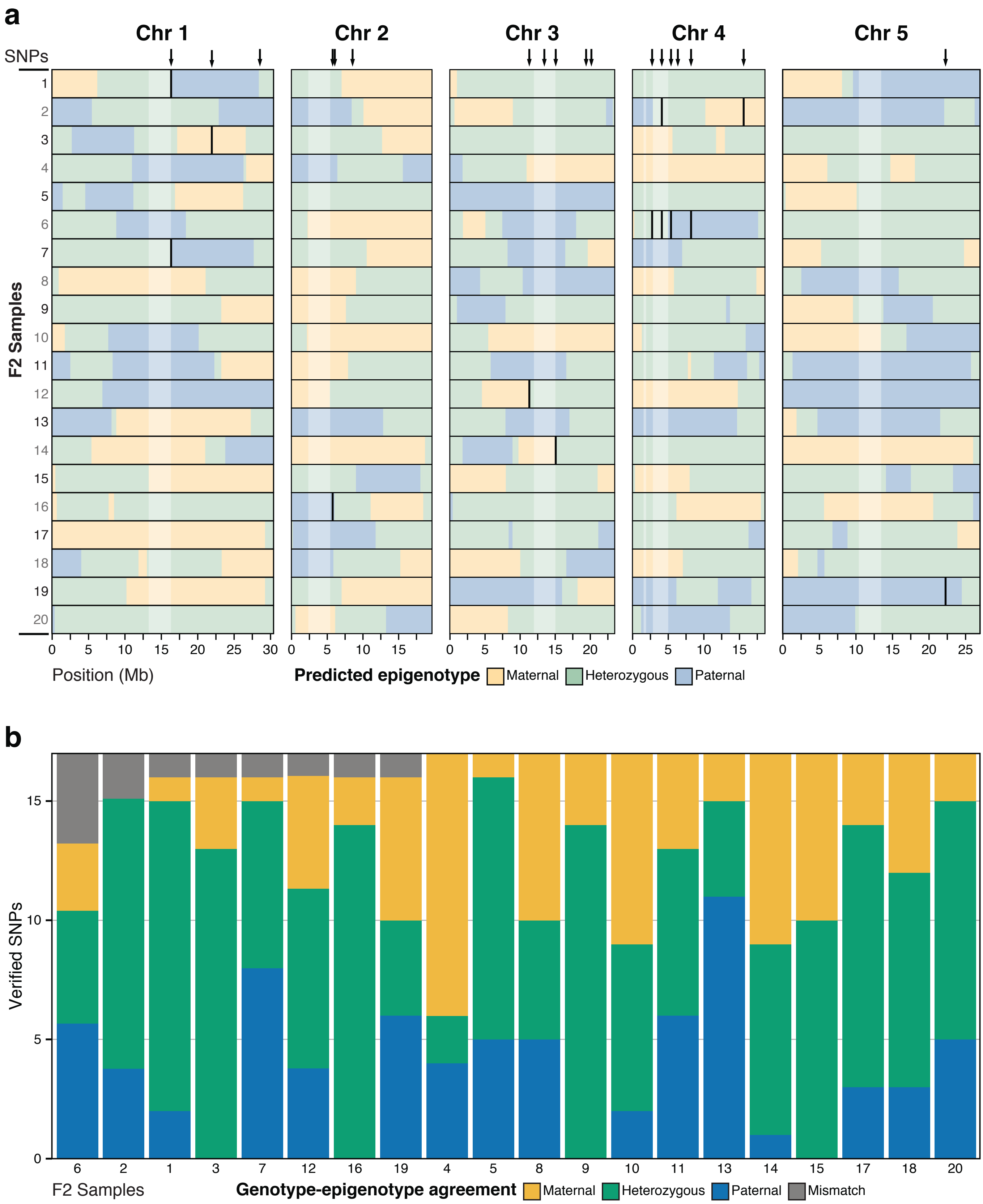

Fig. S9. Full SNP Profile in F2s. (a) Results of SNP analysis comparing predicted epigenotype and predicted genotype from WGBS reads at SNP locations in the parents. Arrows indicate position of SNPs along each chromosome. Black vertical lines denote locations were predicted epigenotype and predicted genotype did not agree. Fill color indicates predicted epigenotype, yellow for maternal homozygous, green for heterozygous, and blue for paternal homozygous. Lighter fill color denotes centromere. (b) Distribution of SNP agreement for each F2 sample. Yellow, green, and blue indicate both methods predicted maternal homozygous, heterozygous, and paternal homozygous, respectively. Gray indicates mismatch between the methods.

Fig. S10

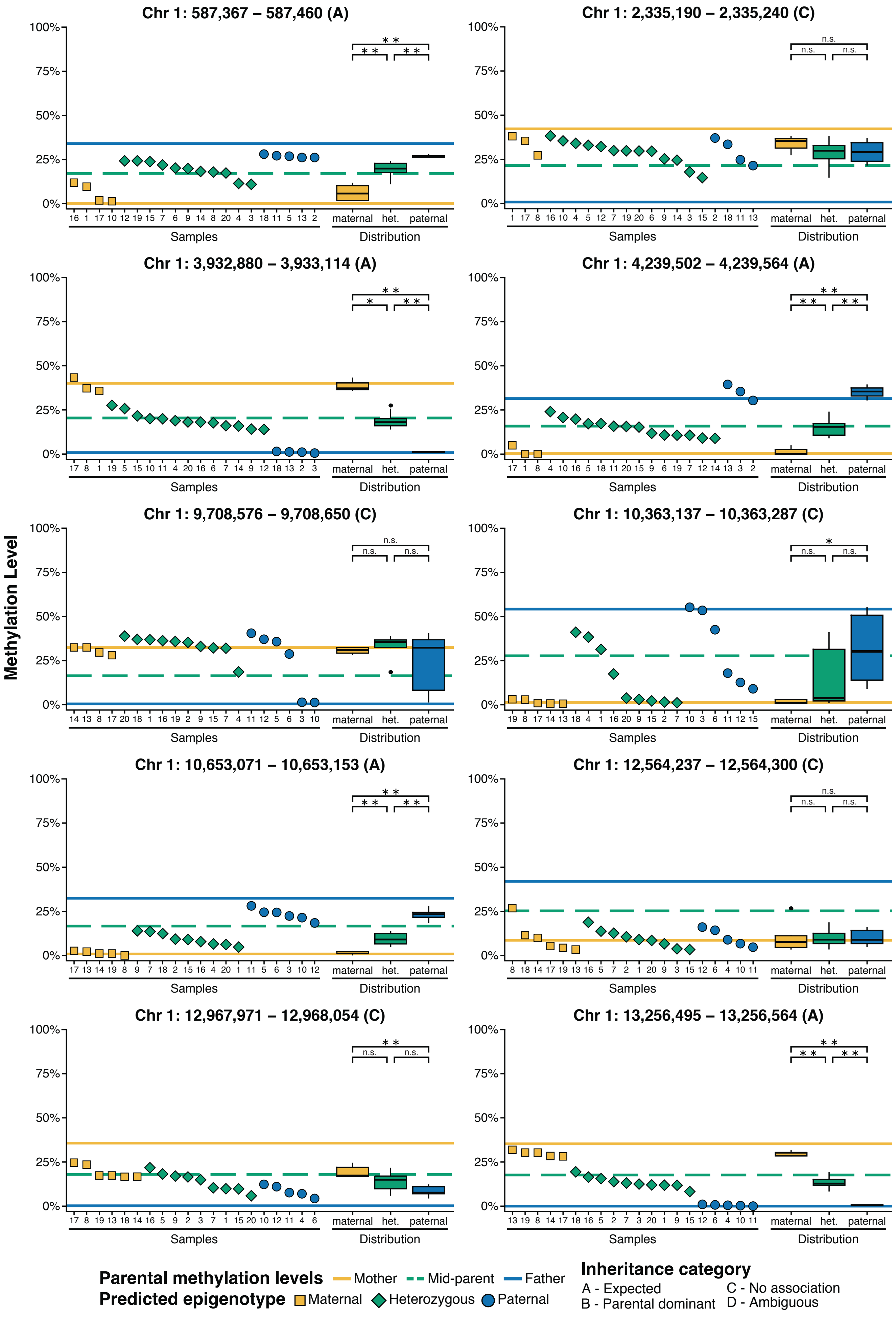

Chr 1: 587,367 – 587,460 (A)
Chr 1: 2,335,190 – 2,335,240 (C)
Chr 1: 3,932,880 – 3,933,114 (A)
Chr 1: 4,239,502 – 4,239,564 (A)
Chr 1: 9,708,576 – 9,708,650 (C)
Chr 1: 10,363,137 – 10,363,287 (C)
Chr 1: 10,653,071 – 10,653,153 (A)
Chr 1: 12,564,237 – 12,564,300 (C)
Chr 1: 12,967,971 – 12,968,054 (C)
Chr 1: 13,256,495 – 13,256,564 (A)
Methylation Level
Samples
Distribution
maternal
het.
paternal
n.s.
Parental methylation levels
Mother
Mid-parent
Father
Predicted epigenotype
Maternal
Heterozygous
Paternal
Inheritance category
A - Expected
B - Parental dominant
C - No association
D - Ambiguous

Fig. S10

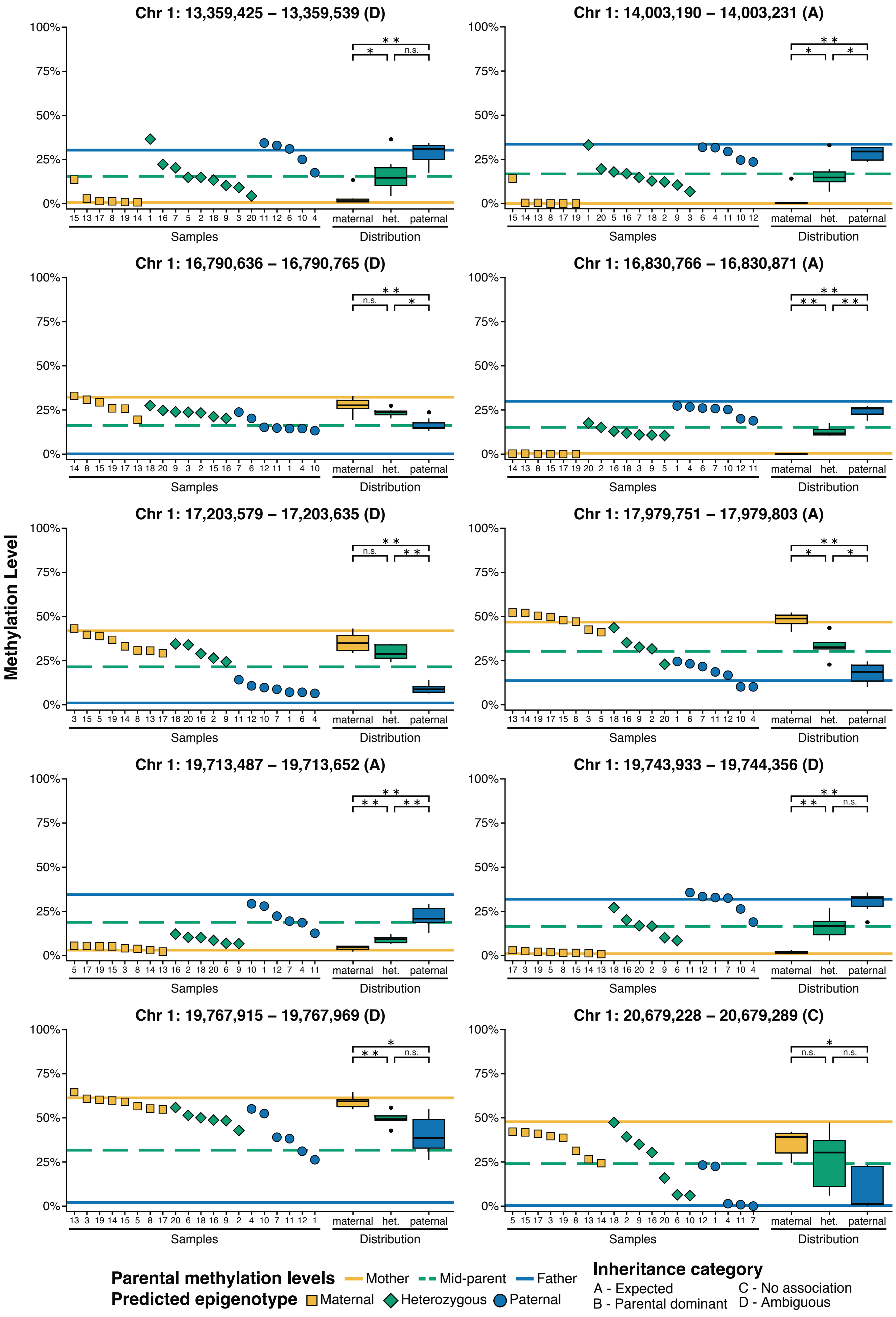
Chr 1: 13,359,425 – 13,359,539 (D)
Chr 1: 14,003,190 – 14,003,231 (A)
Chr 1: 16,790,636 – 16,790,765 (D)
Chr 1: 16,830,766 – 16,830,871 (A)
Chr 1: 17,203,579 – 17,203,635 (D)
Chr 1: 17,979,751 – 17,979,803 (A)
Chr 1: 19,713,487 – 19,713,652 (A)
Chr 1: 19,743,933 – 19,744,356 (D)
Chr 1: 19,767,915 – 19,767,969 (D)
Chr 1: 20,679,228 – 20,679,289 (C)
Methylation Level
100%
75%
50%
25%
0%
Samples
Distribution
maternal
het.
paternal
n.s.
Parental methylation levels
Mother
Mid-parent
Father
Predicted epigenotype
Maternal
Heterozygous
Paternal
Inheritance category
A - Expected
B - Parental dominant
C - No association
D - Ambiguous

Fig. S10

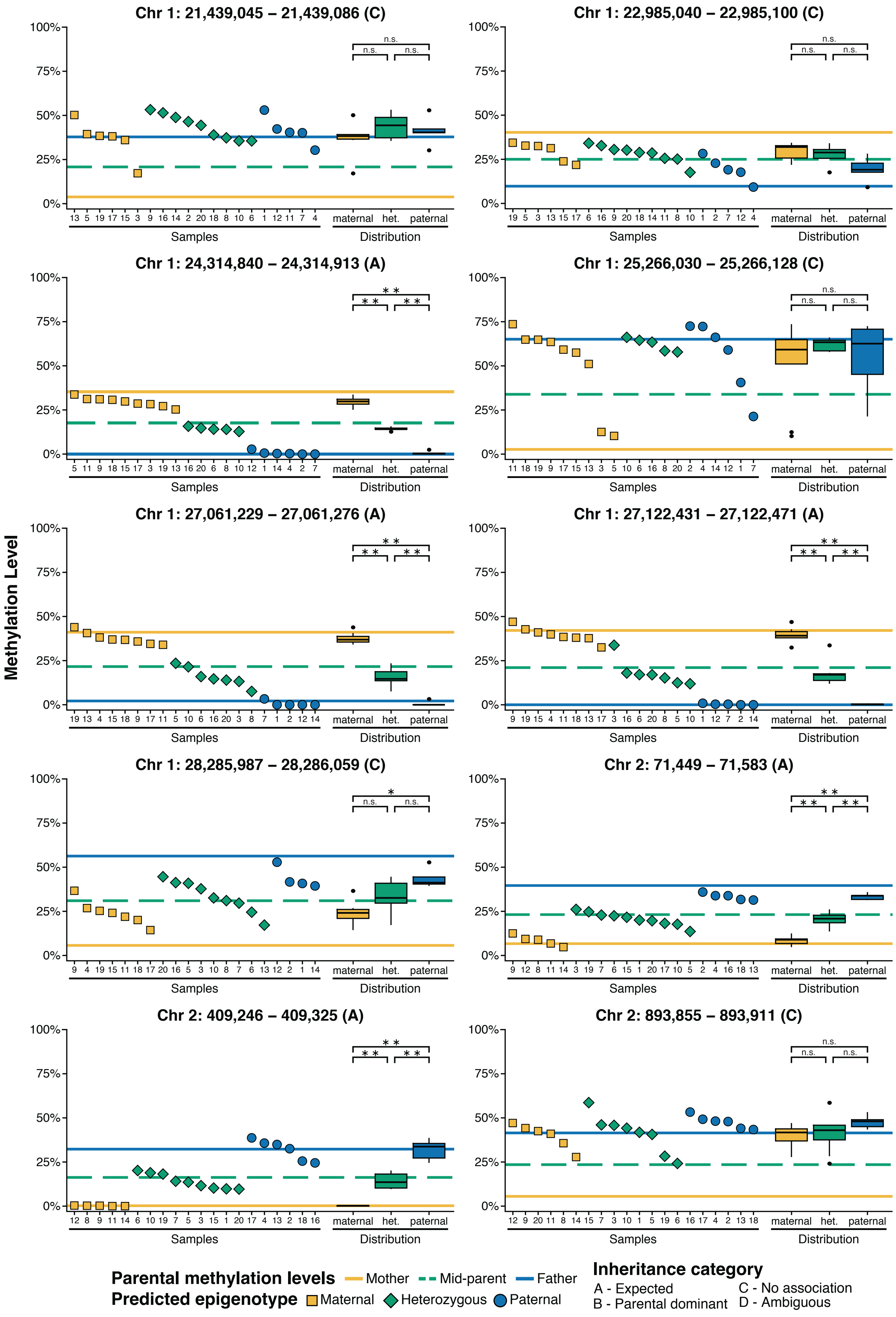
Chr 1: 21,439,045 – 21,439,086 (C)
Chr 1: 22,985,040 – 22,985,100 (C)
Chr 1: 24,314,840 – 24,314,913 (A)
Chr 1: 25,266,030 – 25,266,128 (C)
Chr 1: 27,061,229 – 27,061,276 (A)
Chr 1: 27,122,431 – 27,122,471 (A)
Chr 1: 28,285,987 – 28,286,059 (C)
Chr 2: 71,449 – 71,583 (A)
Chr 2: 409,246 – 409,325 (A)
Chr 2: 893,855 – 893,911 (C)
Methylation Level
100%
75%
50%
25%
0%
maternal
het.
paternal
n.s.
Samples
Distribution
Parental methylation levels
Mother
Mid-parent
Father
Predicted epigenotype
Maternal
Heterozygous
Paternal
Inheritance category
A - Expected
B - Parental dominant
C - No association
D - Ambiguous

Fig. S10

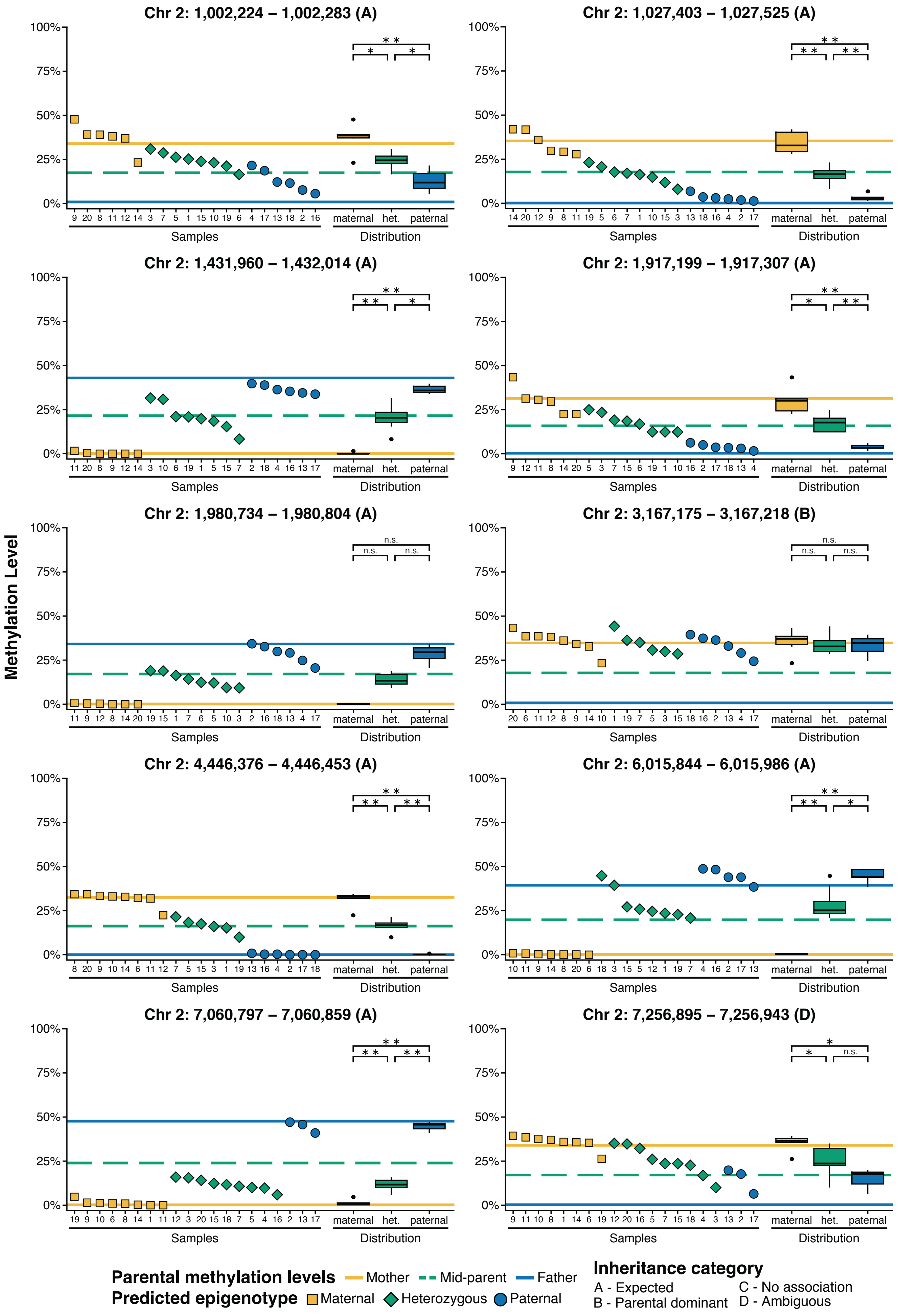
Chr 2: 1,002,224 – 1,002,283 (A)
Chr 2: 1,027,403 – 1,027,525 (A)
Chr 2: 1,431,960 – 1,432,014 (A)
Chr 2: 1,917,199 – 1,917,307 (A)
Chr 2: 1,980,734 – 1,980,804 (A)
Chr 2: 3,167,175 – 3,167,218 (B)
Chr 2: 4,446,376 – 4,446,453 (A)
Chr 2: 6,015,844 – 6,015,986 (A)
Chr 2: 7,060,797 – 7,060,859 (A)
Chr 2: 7,256,895 – 7,256,943 (D)
Methylation Level
100%
75%
50%
25%
0%
maternal
het.
paternal
Samples
Distribution
n.s.
Parental methylation levels
Mother
Mid-parent
Father
Predicted epigenotype
Maternal
Heterozygous
Paternal
Inheritance category
A - Expected
B - Parental dominant
C - No association
D - Ambiguous

Fig. S10

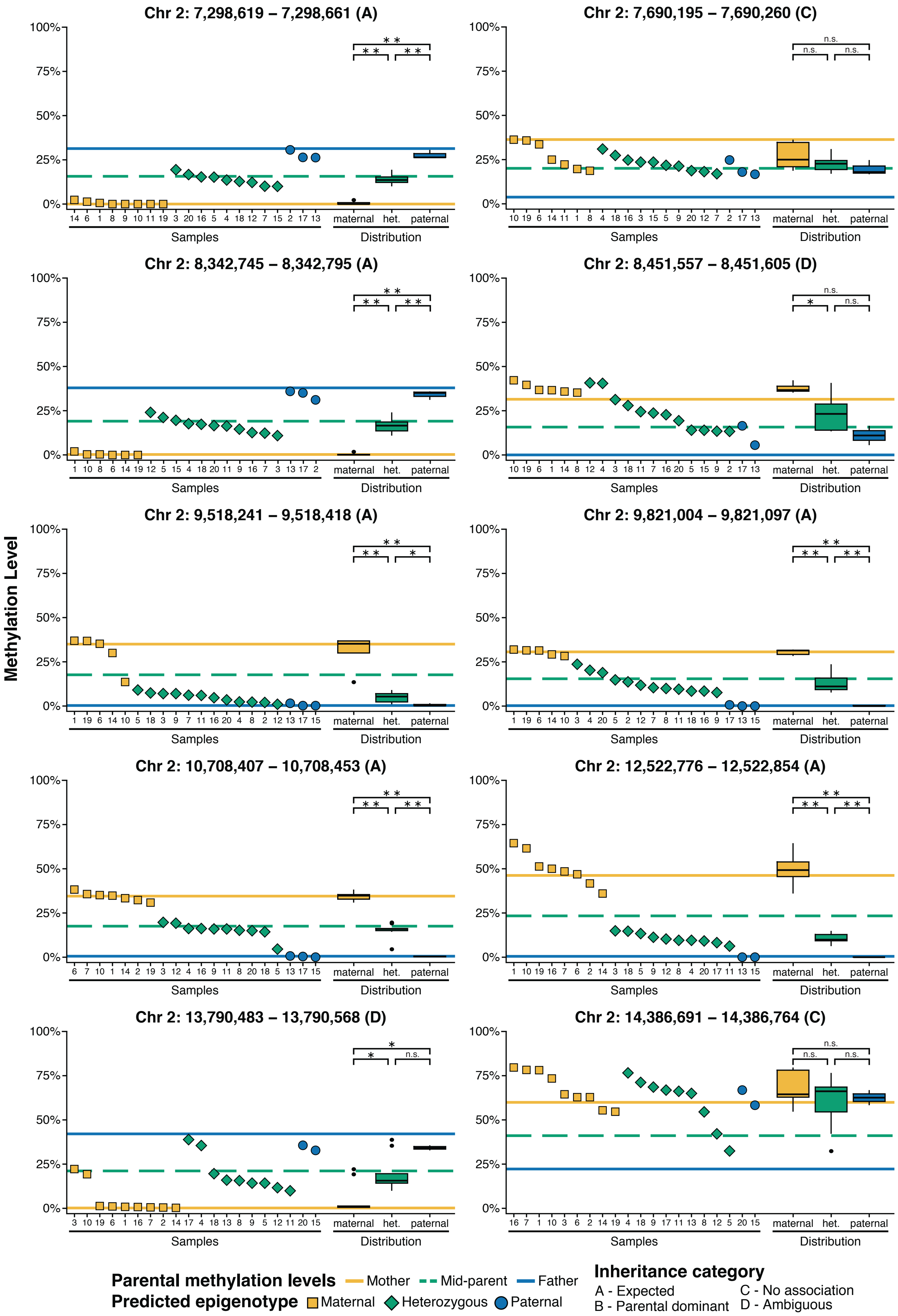

Chr 2: 7,298,619 – 7,298,661 (A)
Chr 2: 7,690,195 – 7,690,260 (C)
Chr 2: 8,342,745 – 8,342,795 (A)
Chr 2: 8,451,557 – 8,451,605 (D)
Chr 2: 9,518,241 – 9,518,418 (A)
Chr 2: 9,821,004 – 9,821,097 (A)
Chr 2: 10,708,407 – 10,708,453 (A)
Chr 2: 12,522,776 – 12,522,854 (A)
Chr 2: 13,790,483 – 13,790,568 (D)
Chr 2: 14,386,691 – 14,386,764 (C)
Methylation Level
100%
75%
50%
25%
0%
Samples
Distribution
maternal
het.
paternal
n.s.
Parental methylation levels
Mother
Mid-parent
Father
Predicted epigenotype
Maternal
Heterozygous
Paternal
Inheritance category
A - Expected
B - Parental dominant
C - No association
D - Ambiguous

Fig. S10

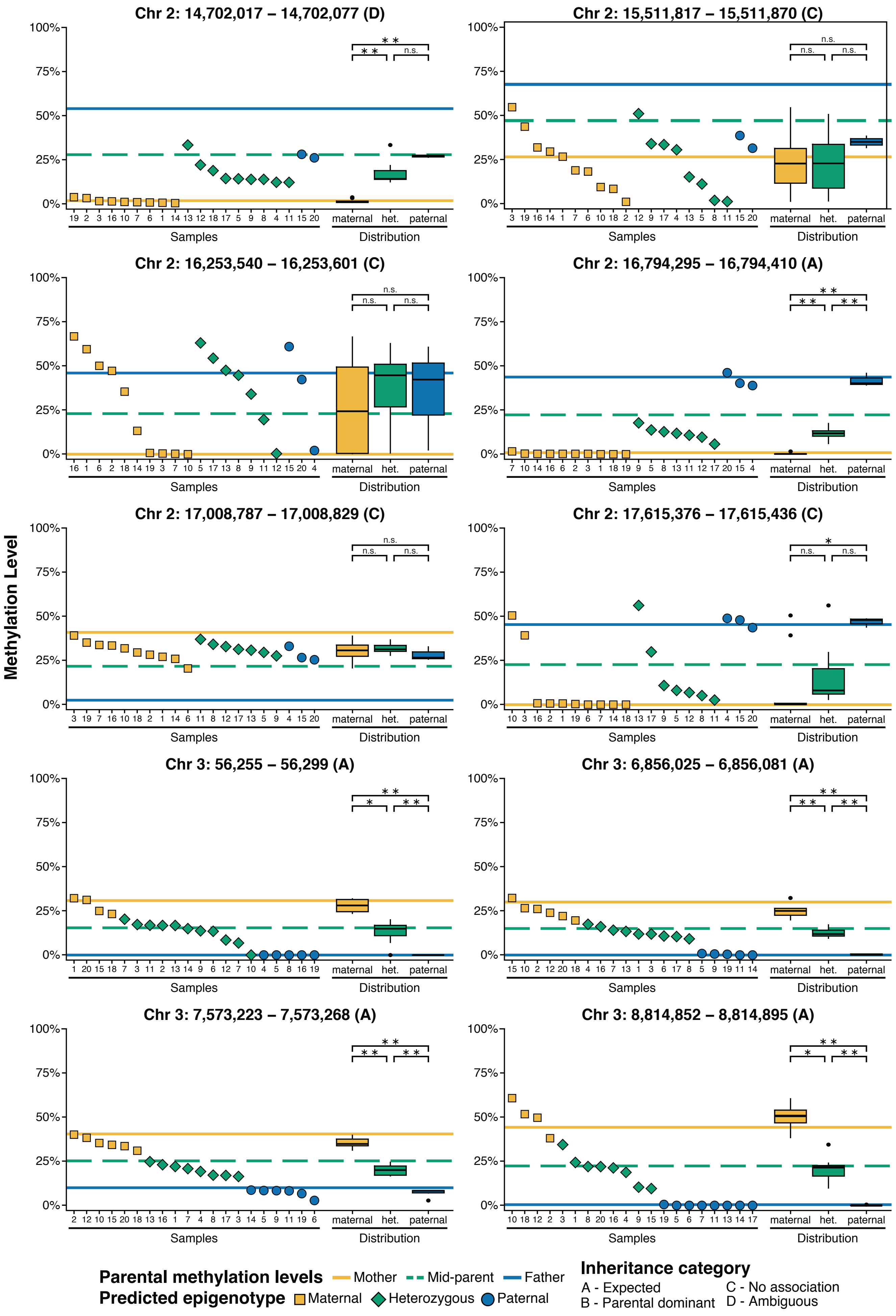
Chr 2: 14,702,017 – 14,702,077 (D)
Chr 2: 15,511,817 – 15,511,870 (C)
Chr 2: 16,253,540 – 16,253,601 (C)
Chr 2: 16,794,295 – 16,794,410 (A)
Chr 2: 17,008,787 – 17,008,829 (C)
Chr 2: 17,615,376 – 17,615,436 (C)
Chr 3: 56,255 – 56,299 (A)
Chr 3: 6,856,025 – 6,856,081 (A)
Chr 3: 7,573,223 – 7,573,268 (A)
Chr 3: 8,814,852 – 8,814,895 (A)
Methylation Level
100%
75%
50%
25%
0%
Samples
Distribution
maternal
het.
paternal
n.s.
Parental methylation levels
Mother
Mid-parent
Father
Predicted epigenotype
Maternal
Heterozygous
Paternal
Inheritance category
A - Expected
B - Parental dominant
C - No association
D - Ambiguous

Fig. S10

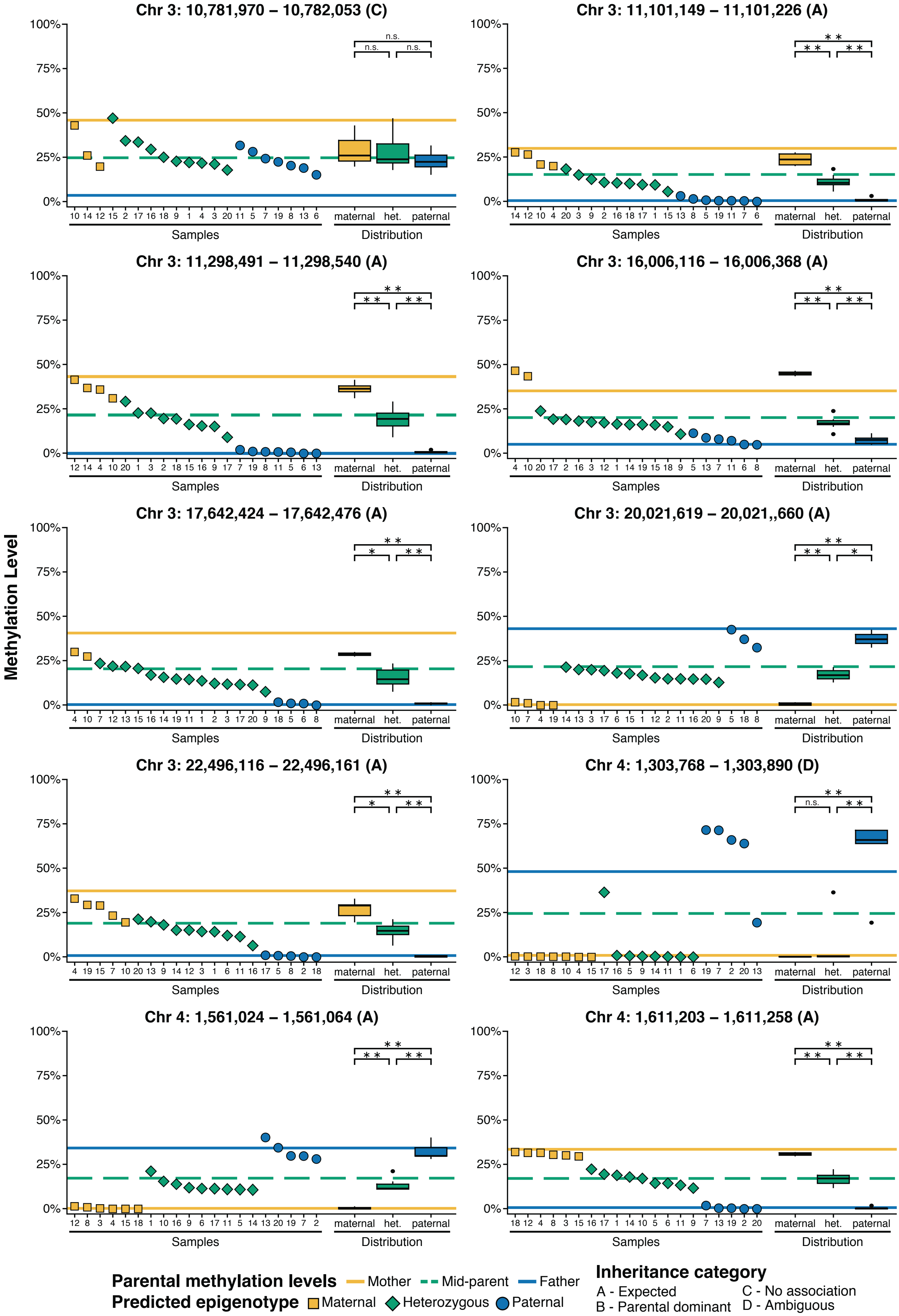
Chr 3: 10,781,970 – 10,782,053 (C)
Chr 3: 11,101,149 – 11,101,226 (A)
Chr 3: 11,298,491 – 11,298,540 (A)
Chr 3: 16,006,116 – 16,006,368 (A)
Chr 3: 17,642,424 – 17,642,476 (A)
Chr 3: 20,021,619 – 20,021,,660 (A)
Chr 3: 22,496,116 – 22,496,161 (A)
Chr 4: 1,303,768 – 1,303,890 (D)
Chr 4: 1,561,024 – 1,561,064 (A)
Chr 4: 1,611,203 – 1,611,258 (A)
Methylation Level
100%
75%
50%
25%
0%
maternal
het.
paternal
Samples
Distribution
n.s.
Parental methylation levels
Mother
Mid-parent
Father
Predicted epigenotype
Maternal
Heterozygous
Paternal
Inheritance category
A - Expected
B - Parental dominant
C - No association
D - Ambiguous

Fig. S10

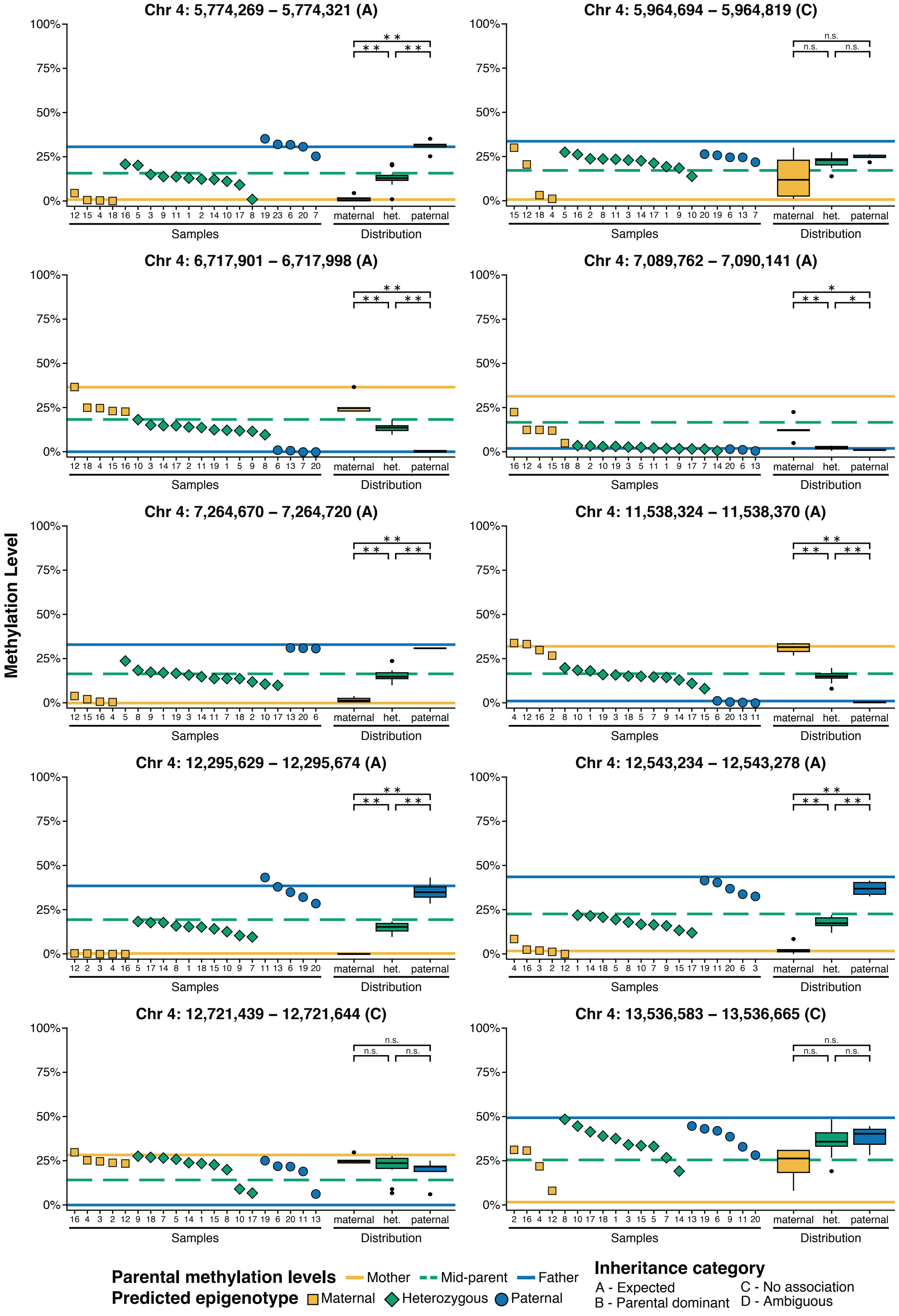
Chr 4: 5,774,269 – 5,774,321 (A)
Chr 4: 5,964,694 – 5,964,819 (C)
Chr 4: 6,717,901 – 6,717,998 (A)
Chr 4: 7,089,762 – 7,090,141 (A)
Chr 4: 7,264,670 – 7,264,720 (A)
Chr 4: 11,538,324 – 11,538,370 (A)
Chr 4: 12,295,629 – 12,295,674 (A)
Chr 4: 12,543,234 – 12,543,278 (A)
Chr 4: 12,721,439 – 12,721,644 (C)
Chr 4: 13,536,583 – 13,536,665 (C)
Methylation Level
100%
75%
50%
25%
0%
Samples
Distribution
maternal
het.
paternal
n.s.
Parental methylation levels
Mother
Mid-parent
Father
Predicted epigenotype
Maternal
Heterozygous
Paternal
Inheritance category
A - Expected
B - Parental dominant
C - No association
D - Ambiguous

Fig. S10

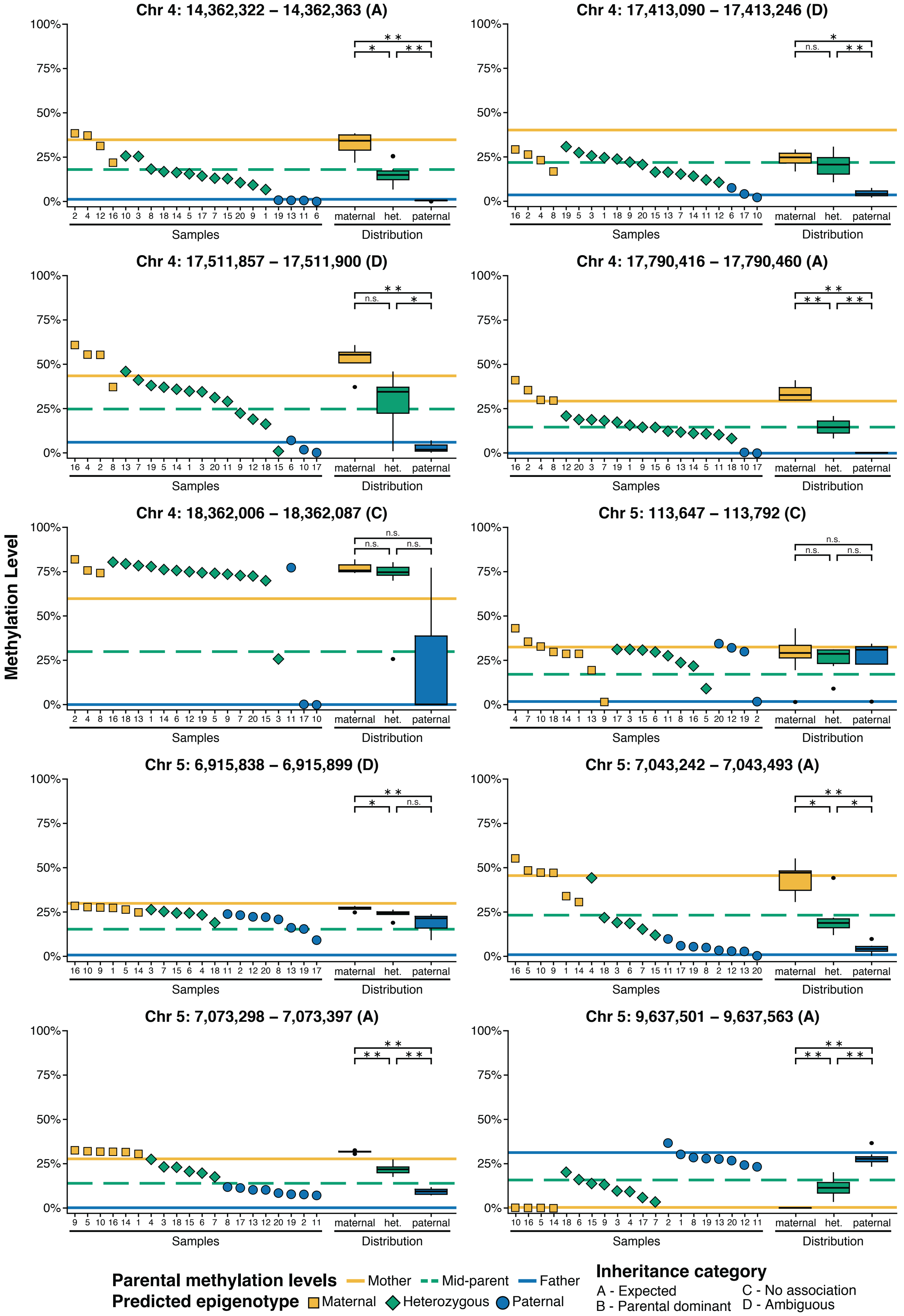
Chr 4: 14,362,322 – 14,362,363 (A)
Chr 4: 17,413,090 – 17,413,246 (D)
Chr 4: 17,511,857 – 17,511,900 (D)
Chr 4: 17,790,416 – 17,790,460 (A)
Chr 4: 18,362,006 – 18,362,087 (C)
Chr 5: 113,647 – 113,792 (C)
Chr 5: 6,915,838 – 6,915,899 (D)
Chr 5: 7,043,242 – 7,043,493 (A)
Chr 5: 7,073,298 – 7,073,397 (A)
Chr 5: 9,637,501 – 9,637,563 (A)
Methylation Level
100%
75%
50%
25%
0%
2 4 12 16 10 3 8 18 14 5 17 7 15 20 9 1 19 13 11 6
16 2 4 8 19 5 3 1 18 9 20 15 13 7 14 11 12 6 17 10
16 4 2 8 13 7 19 5 14 1 3 20 11 9 12 18 15 6 10 17
16 2 4 8 12 20 3 7 19 1 9 15 6 13 14 5 11 18 10 17
2 4 8 16 18 13 1 14 6 12 19 5 9 7 20 15 3 11 17 10
4 7 10 18 14 1 13 9 17 3 15 6 11 8 16 5 20 12 19 2
16 10 9 1 5 14 3 7 15 6 4 18 11 2 12 20 8 13 19 17
16 5 10 9 1 14 4 18 3 6 7 15 11 17 19 8 2 12 13 20
9 5 10 16 14 1 4 3 18 15 6 7 8 17 13 12 20 19 2 11
10 16 5 14 18 6 15 9 3 4 17 7 2 1 8 19 13 20 12 11
maternal het. paternal
Samples
Distribution
n.s.
Parental methylation levels
Mother
Mid-parent
Father
Predicted epigenotype
Maternal
Heterozygous
Paternal
Inheritance category
A - Expected
B - Parental dominant
C - No association
D - Ambiguous

Fig. S10

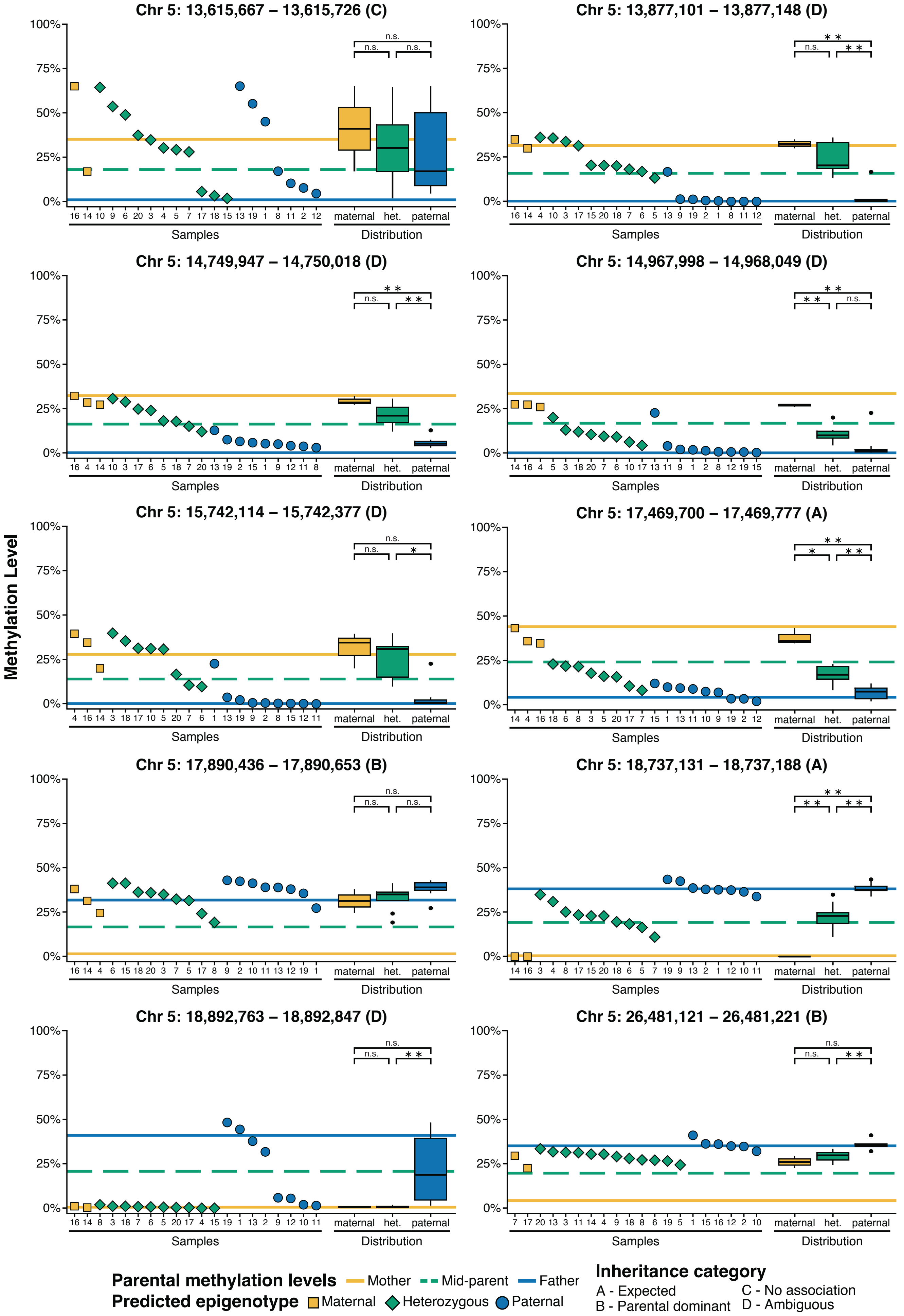
Chr 5: 13,615,667 – 13,615,726 (C)
Chr 5: 13,877,101 – 13,877,148 (D)
Chr 5: 14,749,947 – 14,750,018 (D)
Chr 5: 14,967,998 – 14,968,049 (D)
Chr 5: 15,742,114 – 15,742,377 (D)
Chr 5: 17,469,700 – 17,469,777 (A)
Chr 5: 17,890,436 – 17,890,653 (B)
Chr 5: 18,737,131 – 18,737,188 (A)
Chr 5: 18,892,763 – 18,892,847 (D)
Chr 5: 26,481,121 – 26,481,221 (B)
Methylation Level
100%
75%
50%
25%
0%
maternal
het.
paternal
n.s.
Samples
Distribution
Parental methylation levels
Mother
Mid-parent
Father
Predicted epigenotype
Maternal
Heterozygous
Paternal
Inheritance category
A - Expected
B - Parental dominant
C - No association
D - Ambiguous

Fig. S10. Regions with different epialleles in the parents. Comparison of each F2 methylation level by epigenotype to the parental methylation level and distribution of methylation level for F2s of each epigenotype. For each region, horizontal lines indicated methylation over the region of the mother, father, and calculated mid-parent in yellow, blue, and green, respectively. Point color and shape indicates predicted epigenotype of the F2 at the region. Box plots show distribution of methylation levels for F2 samples grouped by epigenotype. Each region is labeled as expected, parental dominant, no association, and ambiguous based on differences in mean methylation level of F2 samples grouped by epigenotype. * indicates significance at 99%, ** at 99.9%, and n.s. is not significant.

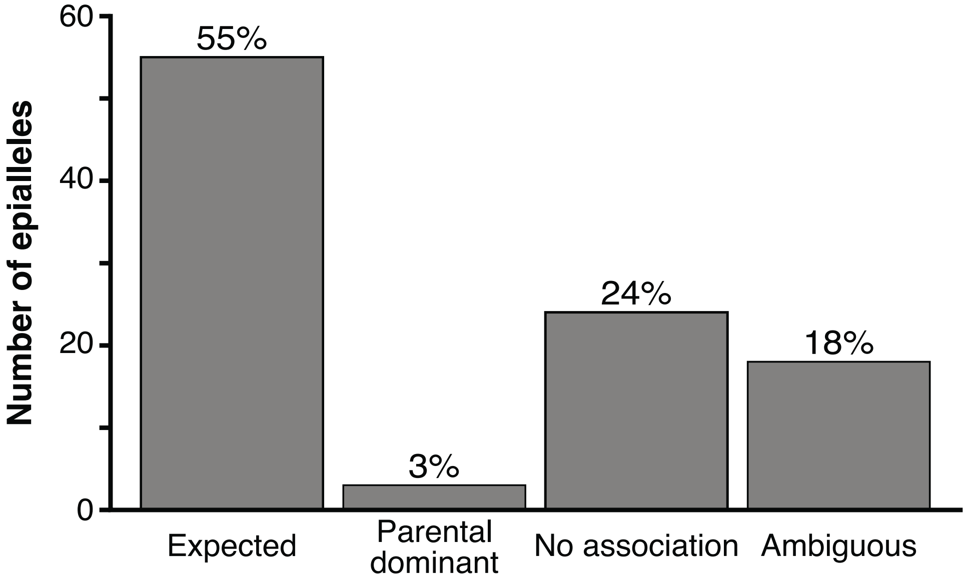

Fig. S11. Distribution of epiallele inheritance categories. Number of epialleles per inheritance category independent of underlying genomic characteristics.
